# Human *CRMP4* mutation and disrupted *Crmp4* expression in mice are associated with ASD characteristics and sexual dimorphism

**DOI:** 10.1038/s41598-017-16782-8

**Published:** 2017-12-01

**Authors:** Atsuhiro Tsutiya, Yui Nakano, Emily Hansen-Kiss, Benjamin Kelly, Masugi Nishihara, Yoshio Goshima, Don Corsmeier, Peter White, Gail E. Herman, Ritsuko Ohtani-Kaneko

**Affiliations:** 1 Institute of Life Innovation Studies, Toyo University, 1-1-1 Itakura, Oura, Gunma, 374-0193 Japan; 2 Graduate School of Life Sciences, Toyo University, 1-1-1 Itakura, Oura, Gunma, 374-0193 Japan; 3 The Institute for Genomic Medicine, Nationwide Children’s Hospital and Department of Pediatrics, The Ohio State University, Columbus, OH 43205 USA; 4 Department of Veterinary Physiology, Graduate School of Agricultural and Life Sciences, The University of Tokyo, 1-1-1 Yayoi, Bunkyo-ku, Tokyo, 113-8657 Japan; 5 Department of Molecular Pharmacology and Neurobiology, Yokohama City University Graduate School of Medicine, 3-9 Fuku-ura, Kanazawa Ward, Yokohama, 236-0004 Japan; 6 Clinical Proteomics and Molecular Medicine, St. Marianna University Graduate School of Medicine, Kawasaki, Japan; 7 Research Center for Biomedical Engineering, Toyo University, 2100 Kujirai, Kawagoe, Saitama, 350-8585 Japan

## Abstract

Autism spectrum disorders (ASD) are more common among boys than girls. The mechanisms responsible for ASD symptoms and their sex differences remain mostly unclear. We previously identified collapsin response mediator protein 4 (*CRMP4*) as a protein exhibiting sex-different expression during sexual differentiation of the hypothalamic sexually dimorphic nucleus. This study investigated the relationship between the sex-different development of autistic features and *CRMP4* deficiency. Whole-exome sequencing detected a *de novo* variant (S541Y) of *CRMP4* in a male ASD patient. The expression of mutated mouse *CRMP4* ^S540Y^, which is homologous to human *CRMP4* ^S541Y^, in cultured hippocampal neurons derived from *Crmp4-*knockout (KO) mice had increased dendritic branching, compared to those transfected with wild-type (WT) *Crmp4*, indicating that this mutation results in altered *CRMP4* function in neurons. *Crmp4*-KO mice showed decreased social interaction and several alterations of sensory responses. Most of these changes were more severe in male *Crmp4*-KO mice than in females. The mRNA expression levels of some genes related to neurotransmission and cell adhesion were altered in the brain of *Crmp4*-KO mice, mostly in a gender-dependent manner. These results indicate a functional link between a case-specific, rare variant of one gene, *Crmp4*, and several characteristics of ASD, including sexual differences.

## Introduction

Since Leo Kanner first reported ‘autistic disturbances of affective contact’ in eight males and three females with symptoms of what would later be called autism^1^, it has been well established that a consistent feature of autism spectrum disorder (ASD) in humans is male predominance. The ratio of males to females ASD is generally around 4:1^2^, although the ratio reported ranges from 1.33:1 to 15.7:1. In addition, the Autism and Developmental Disabilities Monitoring Network conducted a multi-site, population-based study in the United States that revealed an increase in the ratio of males to females among 8-year-old children with ASD over the past 10 years^3^. This ratio increased from 4.25:1 in children born in 1994 to 4.54:1 in children born in 1998 and to 4.65:1 in children born in 2000. To date, hundreds to thousands of genes have been considered as autism candidates or susceptibility genes in autism gene databases^4^. While some of these genes, including SH3 and multiple ankyrin repeat domains 1 (*SHANK1*), retinoic acid-related orphan receptor alpha (*RORA*) and methyl-CpG-binding protein 2 (*MeCP2*), are reported as possibly involved in the male predominance of ASD^5–7^, there is still a lack of definitive information regarding the genes or mechanisms underlying this sex difference. The increasing male to female ratio in ASD may lead to the hypothesis that the regulation of genes related to sex difference in ASD is affected by biological or environmental exposures (e.g. exposure to hormones or endocrine disruptors), making males more susceptible to the disorder^8^, although there may be other possibilities including the expanding diagnostic criteria of ASD, which lead to the ascertainment of a greater number of boys with high functioning autism^9,10^.

Our previous proteomics studies identified collapsin response mediator protein 4 (CRMP4), also called DPYSL3, a member of the CRMP family (CRMP1–5), as a protein exhibiting sex-associated differences in expression during differentiation of the sexually dimorphic nucleus of the hypothalamus^11^. In addition, the expression of *Crmp4* mRNA in the hypothalamic sexually dimorphic nucleus on post-natal day 0 (PD0) was altered in females treated with androgen during the late pre-natal stage^11^, indicating that *Crmp4* expression is sex-steroid dependent in the rat, at least in this specific brain region.

It was previously reported that a missense variant in the *CRMP4* gene was found, in addition to four other *de novo* variants, in an ASD proband from the Simons Simplex Collections^12^. In the present study, we identified another likely pathogenic missense variant in the *CRMP4* gene in a male with ASD using exome sequencing. Although such variants in *CRMP4* appear to be very rare, they may provide clues to the pathways and mechanisms responsible for the male dominance of ASD symptoms. In addition, through the assessment of behaviour as well as gene expression in *Crmp4*-knockout (KO) mice of both sexes, we examined the role of *Crmp4* dysfunction in ASD pathogenesis.

## Results

### *De novo* variant of *CRMP4* (*DPYSL3*) in sporadic autism case

**Figure 1:**
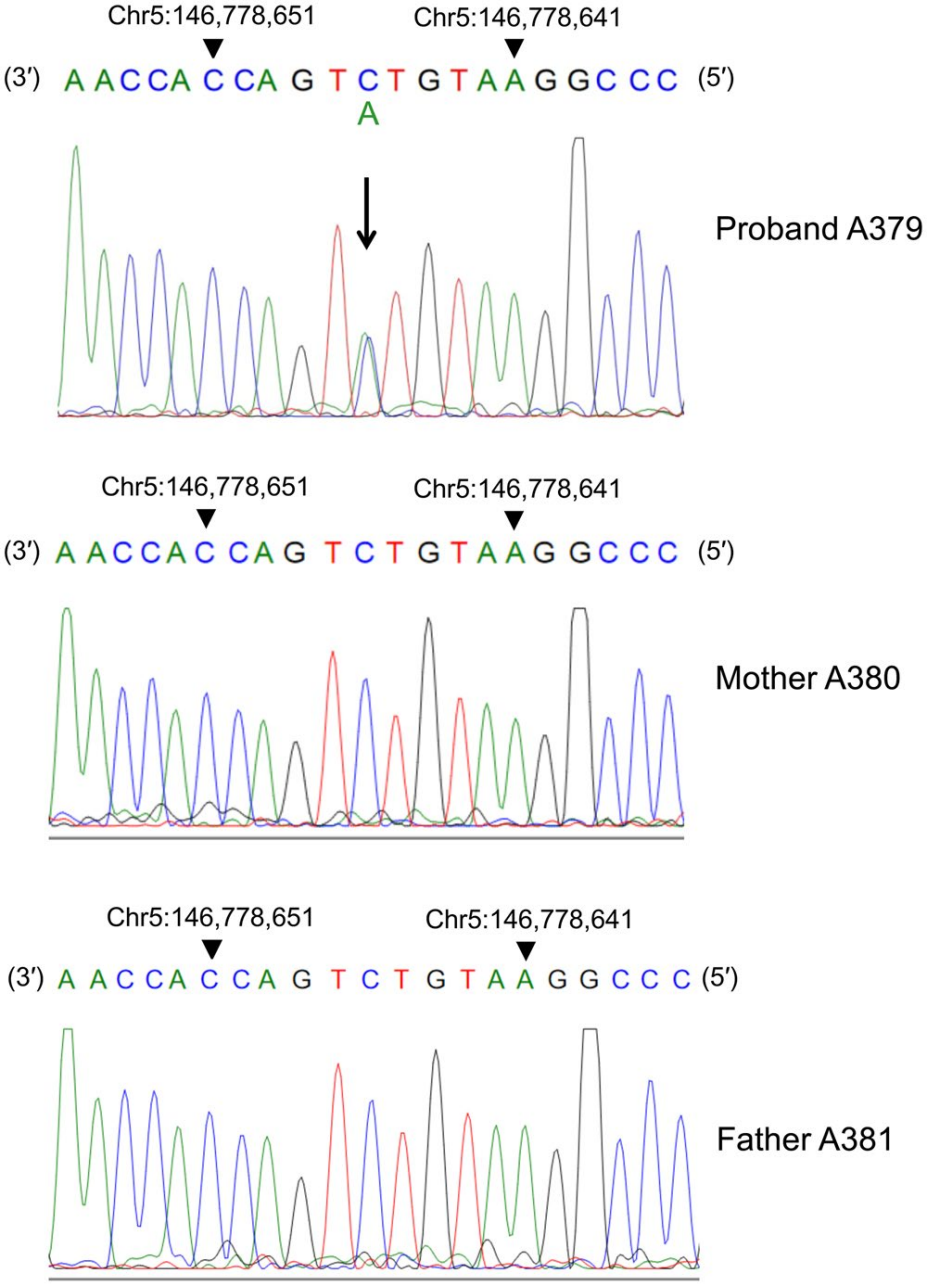
*de novo* variant of *CRMP4* in sporadic autism case. A downward arrow indicates the mutated residue (C > A). This mutation resulted in a change in the amino acid sequence of *CRMP4* (S541Y). Nucleotides are shown in coloured, single-letter codes.

As a part of the efforts to identify genes involved in the pathogenesis of ASD, the Central Ohio Registry for Autism (CORA)^13^ was initiated to enroll isolated cases and multiplex families with one or more children diagnosed with ASD. Detailed clinical phenotyping, including family history and psychological testing, and molecular studies were performed according to the institutional guidelines. We undertook exome sequencing in 72 families in which there appeared to be an isolated, sporadic case of ASD. In patient A379, we detected a *de novo* cytosine to adenine transversion at nucleotide 1622 of the human full length cDNA (c.1622C > A, an arrow in Fig. 1), which leads to the change of a serine to a tyrosine at amino acid position 541 (p.Ser541Tyr) of the CRMP4 protein [NM_001197294.1 (DPYSL3):c.1622C > A:p.(Ser541Tyr); NC_000005.9:g.146778646G > T]. DPYSL3 is also called CRMP-4, CRMP4, dihydropyrimidinase-related protein 3 (DRP-3, DRP3), LCRMP, TUC4, Unc-33-like phosphoprotein, ULIP and Ulip-1. In the patient, no other damaging *de novo* variants were found. The C to A transition in *CRMP4* was not present in the parents (Fig. 1) or siblings (data not shown).

The variant is in a highly conserved region of the predicted protein (GERP++5.47)^14^ and is predicted borderline deleterious by SIFT (0.07)^15^ and probably damaging by Polyphen2 (0.992)^16^. Further, it is predicted to be deleterious by both of the ensemble-based scores MetaSVM and MetaLR (0.142 and 0.566, respectively)^17^, which integrate additional functional prediction and conservation scores, as well as allele frequency. This rare variant is not reported in either the Exome Aggregation Consortium (ExAC) database or the Genome Aggregation Database (gnomAD). The Human Splicing Finder predicts the creation of an exonic splicing silencer and potential alteration of splicing. Both the adaptive boosting (ADA) and random forest (RF) method scores, from the database of human SNVs within splicing consensus regions (dbNSFP-dbscSNV), also predict splice alteration (0.977 and 0.712, respectively; PMID: 25416802).

### Effect of *Crmp4*-KO, *Crmp4* and *Crmp4*^S540Y^ on the dendritic morphology of cultured hippocampal neurons

**Figure 2:**
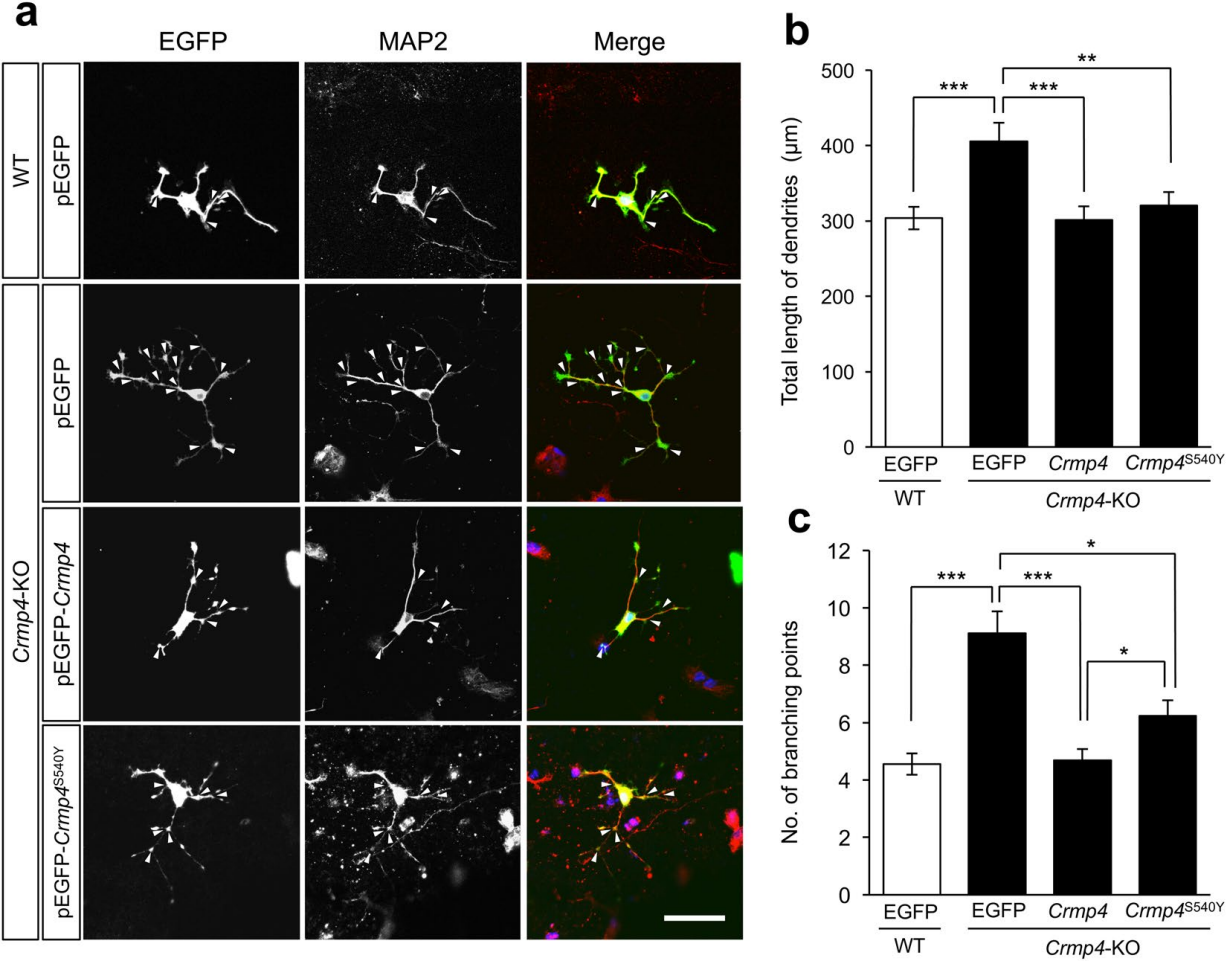
Effect of *Crmp4* or *Crmp4* ^S540Y^ gene expression on the dendritic arborisation of cultured hippocampal cells. S540Y mutation in mouse *Crmp4* gene is homologous to S541Y in human *CRMP4* gene found in the ASD patient. Representative images of cultured hippocampal cells from wild-type (WT) mice transfected with control (pEGFP) vector, those from *Crmp4*-KO mice transfected with pEGFP vector, those from *Crmp4*-KO mice transfected with pEGFP-*Crmp4* vector, and those from *Crmp4*-KO mice transfected with pEGFP-*Crmp4* ^S540Y^ (**a**). Cultured cells were fixed at DIV-3 (3 days *in vitro*) with 4% paraformaldehyde and then immunocytochemically stained with antibodies against MAP2 (red) and EGFP (green). Nuclei were stained with Höechst 33258 (blue). The total length of MAP2- and EGFP-double labelled dendrites and the number of the dendritic branching (arrow heads) were measured for each double labelled neuron. Scale bar: 50 µm. Bar graphs showing the average of total dendritic length (**b**) and the average of the number of dendritic branches (**c**). *n* = 81, 71, 82, and 61 for WT neurons transfected with pEGFP, *Crmp4*-KO neurons transfected with pEGFP, *Crmp4*-KO neurons transfected with pEGFP-*Crmp4*, and *Crmp4*-KO neurons transfected with pEGFP-*Crmp4* ^S540Y^, respectively, from at least three independent experiments. Bars indicate mean ± SEM. Asterisks indicate statistical significance (*p < 0.05, ** *p* < 0.01, *** *p* < 0.001; one-way ANOVA followed by Fisher’s PLSD *post*-*hoc* tests).

We and others have previously reported that the deficiency of *Crmp4* affects dendritic morphology^18,19^. In addition, there have been several recent reports demonstrating altered dendritic development in ASD mouse models^20,21^, leading us to focus on dendritic development in the present study. To examine the functional consequences of the missense variant found in the ASD patient, we studied the dendritic arborisation in cultured hippocampal neurons which are routinely used for analysis of dendrites. In this study, primary hippocampal neurons from male animals were used to assess the general effects of *Crmp4* or *Crmp4* ^S540^ expressions on dendritic growth. We compared WT hippocampal neurons with *Crmp4*-KO hippocampal neurons transfected with a construct expressing *Crmp4* ^S540Y^ that contains a point mutation in the homologous site of S541 in human *CRMP4* or WT *Crmp4* (Fig. 2a).

As shown in Fig. 2b and c, the total length of dendrites and the number of branching points (arrowheads in Fig. 2a) were significantly increased in neurons derived from *Crmp4*-KO mice transfected with the control pEGFP vector than in those derived from WT mice transfected with the same control vector or in those derived from *Crmp4*-KO mice transfected with pEGFP-*Crmp4* (*p* < 0.001). The total dendritic length of each double-labelled neuron derived from *Crmp4*-KO mice transfected with pEGFP-*Crmp4* ^S540Y^ did not differ significantly from those derived from *Crmp4*-KO mice transfected with pEGFP-*Crmp4* and those derived from WT mice transfected with pEGFP (Fig. 2b). However, neurons derived from *Crmp4-*KO mice transfected with pEGFP-*Crmp4* ^S540Y^ showed significantly greater numbers of dendritic branching points than those derived from *Crmp4*-KO mice transfected with pEGFP-*Crmp4* (Fig. 2c, *p* < 0.05), although they had less branching points than the neurons derived from *Crmp4*-KO mice transfected with pEGFP (*p* < 0.05). These cell culture studies demonstrate that the S540Y mutation in the mouse *Crmp4* gene, which is homologous to S541Y in the human *CRMP4* gene, results in a partially reduced CRMP4 function in suppressing dendritic branching.

### *Crmp4*-KO mice demonstrate normal activities and responses in the open-field test, elevated plus maze, and novel object recognition test

**Figure 3:**
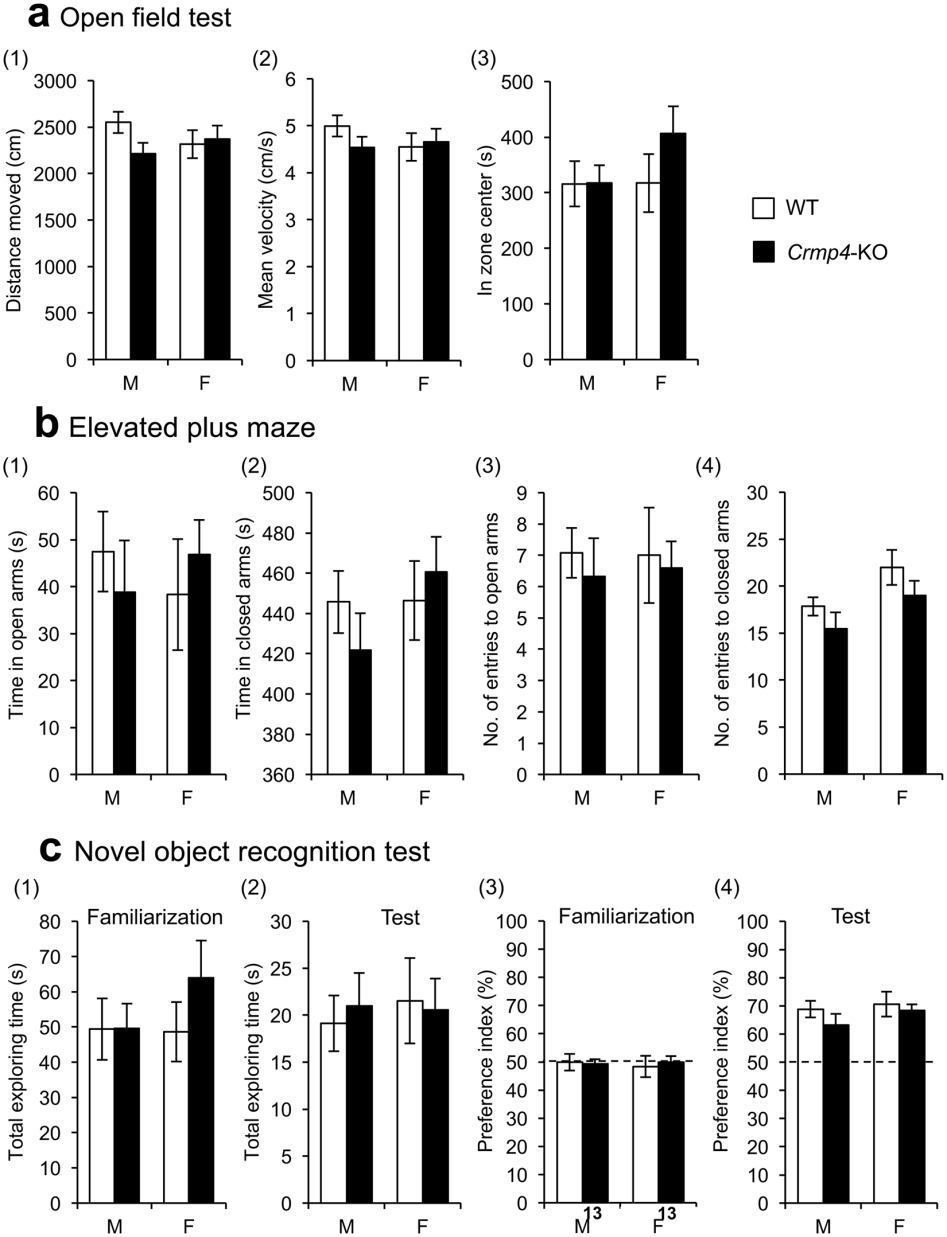
Behavioural phenotypes of wild-type (WT) and *Crmp4*-KO mice. (**a**) Open-field test for 20 min with total distance moved (1), mean velocity (2) and total time spent in the central zone (3). Male WT, n = 17; male *Crmp4*-KO, n = 15; female WT, n = 12; and female *Crmp4*-KO, n = 14. (**b**) Elevated plus maze (distance from floor = 60 cm) comprising two open arms (27.5 × 4.5 cm) and two closed arms made of clear Plexiglas (27.5 × 4.5 × 14 cm) extending from a central (4.5 × 4.5 cm) platform for 10 min. The time spent in open (1) and closed (2) arms and the number of entries to open (3) and closed (4) arms in the elevated plus maze test. Male WT, n = 11; male *Crmp4*-KO, n = 10; female WT, n = 9; and female *Crmp4*-KO, n = 12. (**c**) Novel object recognition test to assess memory acquisition and retention performance. Total duration of exploring the objects during familiarisation (1) and test (2) phases of the novel object recognition test. Preference index, which expresses the ratio of the amount of time spent exploring novel object during familiarisation (3) and test (4) phases. Male WT, n = 13; male *Crmp4*-KO, n = 17; female WT, n = 13; and female *Crmp4*-KO, n = 13. Values are expressed as mean ± SEM.

**Figure 4:**
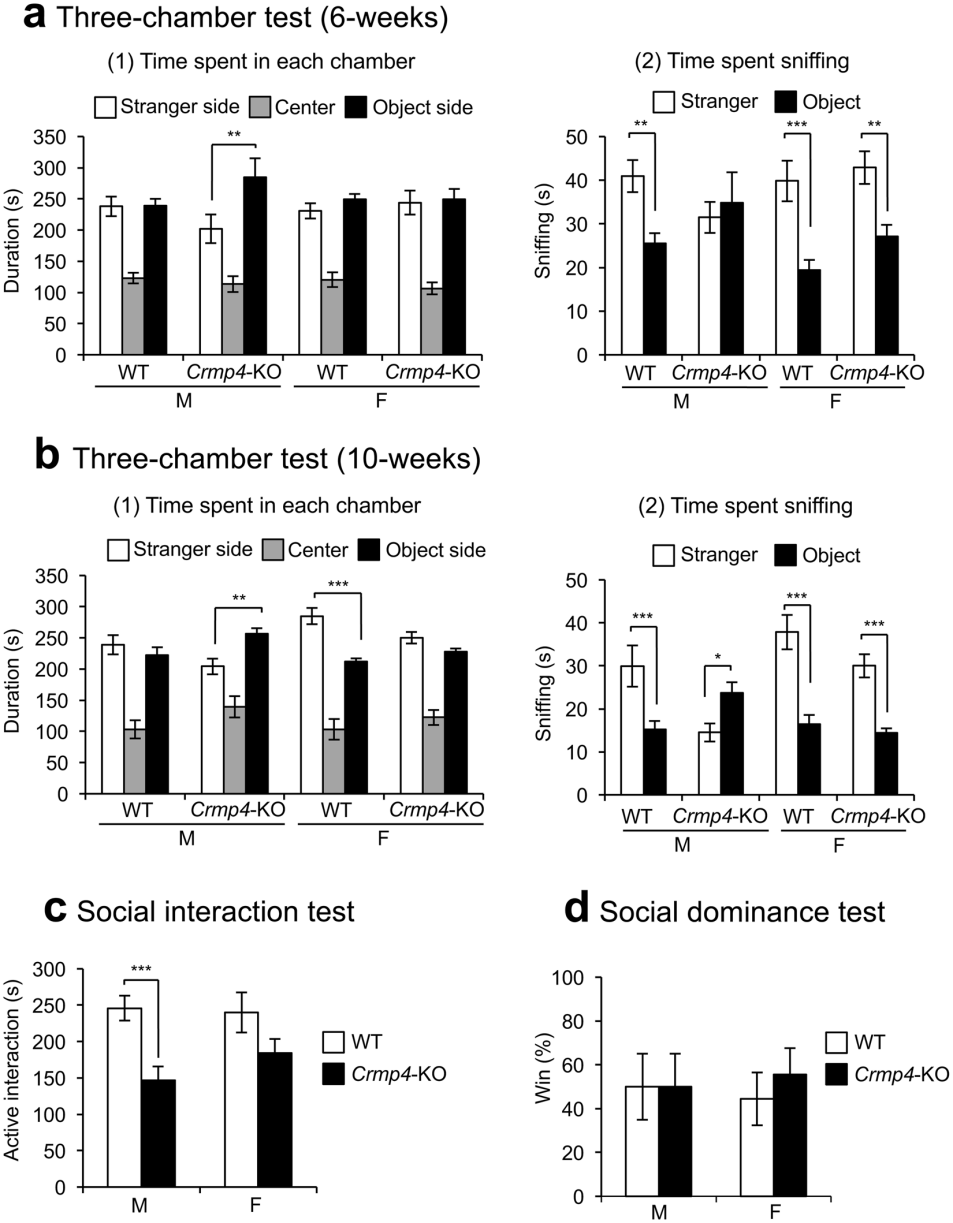
Social behaviours in wild-type (WT) and *Crmp4*-KO mice. (**a** and **b**) The three-chamber test performed at 6-weeks [(**a)**, male WT, n = 11; male *Crmp4*-KO, n = 10; female WT, n = 11; and female *Crmp4*-KO, n = 12] and 10-weeks [(**b)**, male WT, n = 10; male *Crmp4*-KO, n = 8; female WT, n = 9; and female *Crmp4*-KO, n = 11]. The testing apparatus comprised two sides and one centre chamber (19 × 40 × 22 cm, each) divided by two doors (11.5 cm wide). An age-matched unfamiliar C57BL/6N WT mouse of the same sex (stranger) enclosed in a wire cage (φ82 × 93.1 mm) was placed behind the partition in one side chamber (stranger side chamber) and an empty wire cage (novel object) was placed behind the partition in the other side chamber (object side chamber) of the three-chambered apparatus. Time spent in each chamber (1) and time spent sniffing stranger and novel object (2). (**c**) Social interaction test to assess active interaction time in 10 min. Male WT, n = 15; male *Crmp4*-KO, n = 17; female WT, n = 12; and female *Crmp4*-KO, n = 17. (**d**) The tube test to show the difference in social dominance between WT and *Crmp4*-KO mice. An age-matched pair of unfamiliar mice (one WT and one *Crmp4*-KO) of the same sex and similar body weight (within 5% difference) was placed into opposite ends of a clear acrylic tube and simultaneously released. The test ended when one mouse completely retreated from the tube, who was assigned a score of zero (loser). The remaining mouse was assigned a score of one (winner). Male WT, n = 6; male *Crmp4*-KO, n = 6; female WT, n = 8; and female *Crmp4*-KO, n = 8. Values are expressed as mean ± SEM. *p < 0.05, **p < 0.01, ***p < 0.001 (three-way ANOVA followed by Fisher’s PLSD *post*-*hoc* tests).

To examine the general characteristics of *Crmp4-*KO mice, KO mice and their WT littermates were subjected to several behavioural tests. Specifically, we focused on behaviors in young or adolescent mice because we speculated that early behavioral deficits in these *Crmp4*-KO mice might link better to ASD-like phenotypes that are apparent in childhood. Testing was performed sequentially on the same cohort of animals at approximately one week intervals with the least invasive (upsetting) tests performed first. Initially, open field tests were performed at 4 weeks of age. Two-way ANOVA revealed that there were no significant differences by genotype and sex and no interaction between these two factors (genotype × sex) in the distance moved (Fig. 3a-(1)), velocity (Fig. 3a-(2)), or time spent in the central region (Fig. 3a-(3)) in the open-field test. Elevated plus maze test was further performed at 4 weeks of age to assess the anxiety levels of the mice. Data showed no significant differences regarding the time spent in open (Fig. 3b-(1)) and closed (Figs. 3b-(2)) arms and in the number of entries to open (Fig. 3b-(3)) and closed (Fig. 3b-(4)) arms. These results suggest that *Crmp4*-KO mice of both sexes have normal locomotive activity and anxiety levels as assessed by the testing modality.

The novel object recognition test at 5 weeks of age showed that the total exploration time in the familiarisation phase (Fig. 3c-(1)) as well as in the test phase (Fig. 3c-(2)) was not significantly different between genotypes and sexes and that there was no interaction between these two factors (two-way ANOVA). These data indicate that exploration behaviour towards inanimate objects is unaffected by *Crmp4* deletion in both sexes, although female *Crmp4*-KO mice tended to spend more time exploring during the familiarisation phase (Fig. 3c-(1)). In addition, the amount of time spent exploring the same objects (‘I’ and ‘II’ in Supplementary Fig. S1) did not differ between genotypes nor between sexes during the familiarisation phase (Fig. 3c-(3)). In the test phase, WT and *Crmp4*-KO mice of both sexes spent more time exploring the novel object (denoted by ‘III’ in Supplementary Fig. S1) than the familiar one (Fig. 3c-(4)), with no significant differences in the time between genotypes and sexes. These results indicate normal abilities of *Crmp4*-KO mice of both sexes in short-term memory acquisition.

The precise behavioural data and their results from statistical analysis are all shown in Supplementary Tables S1 and S2.

### Male *Crmp4*-KO mice demonstrate decreased social behaviour in social contact but not in social dominance

The three-chamber test at 6 weeks of age revealed that only *Crmp4*-KO males stayed for a significantly longer time in the ‘object side chamber’ than in the ‘stranger side chamber’ (Fig. 4a-(1), *p* < 0.01). WT mice of both sexes and *Crmp4*-KO females spent more time sniffing the stranger mouse than the novel object (Fig. 4a-(2)). These differences are significant by *post hoc* PSDL test, but the differences in male WT and female *Crmp4*-KO mice did not survive correction for multiple testing, such as Bonferroni correction (for *p*-values from multiple comparison, see Supplementary table S2). The time spent sniffing the novel object versus the stranger mouse was not significantly different in *Crmp4*-KO males (Fig. 4a-(2)).

Since the three-chamber test at 6 weeks of age suggested possible differences between genotypes, we further performed the same experiment using a separate cohort of older mice (10 weeks of age, Fig. 4b, Supplementary Table S1, and S2). The results showed that WT females spent a significantly longer time in the ‘stranger side chamber’ than in the ‘object side chamber’ (Fig. 4b-(1), *p* < 0.001), whereas *Crmp4*-KO males spent a significantly longer time in the ‘object side chamber’ than in the ‘stranger side chamber’ (Fig. 4b-(1), *p* < 0.01). The time sniffing the stranger mouse was significantly longer than that sniffing the novel object in WT mice of both sexes and in *Crmp4*-KO females (Fig. 4b-(2), *p* < 0.001). Conversely, *Crmp4*-KO males spent a significantly longer time sniffing the novel object than the stranger mouse (Fig. 4b-(2), *p* < 0.05).

We further investigated social behaviours of *Crmp4*-KO mice using social interaction test at 6 weeks of age (Fig. 4c). *Crmp4*-KO mice spent a significantly shorter time in active interaction than WT littermates (WT and *Crmp4*-KO, 243.28 ± 13.75 and 169.74 ± 13.03 s, respectively), as determined by two-way ANOVA. *Post-hoc* comparison revealed that *Crmp4*-KO males displayed significantly less active interaction than WT males (*p* < 0.001, Fig. 4c, left bars); however, *Crmp4*-KO females did not differ significantly from WT females in active interaction (Fig. 4c, right bars). The results obtained from the three-chamber and social interaction tests indicate a decreased social behaviour in *Crmp4*-KO males.

The tube test was performed at 6 weeks of age to examine social dominance (Fig. 4d). Both WT and *Crmp4*-KO mice had equal winners, indicating that the decreased social activities observed in *Crmp4*-KO mice are unlikely to be due to altered social dominance. It is also unlikely that the decreased social interaction in *Crmp4*-KO males arose from an aversion to novelty because social dominance and exploration towards novelty did not differ between WT and *Crmp4*-KO mice of either sex (Figs 3c and 4d).

### *Crmp4*-KO mice exhibit normal olfactory function at 7 and 11 weeks of age

**Figure 5:**
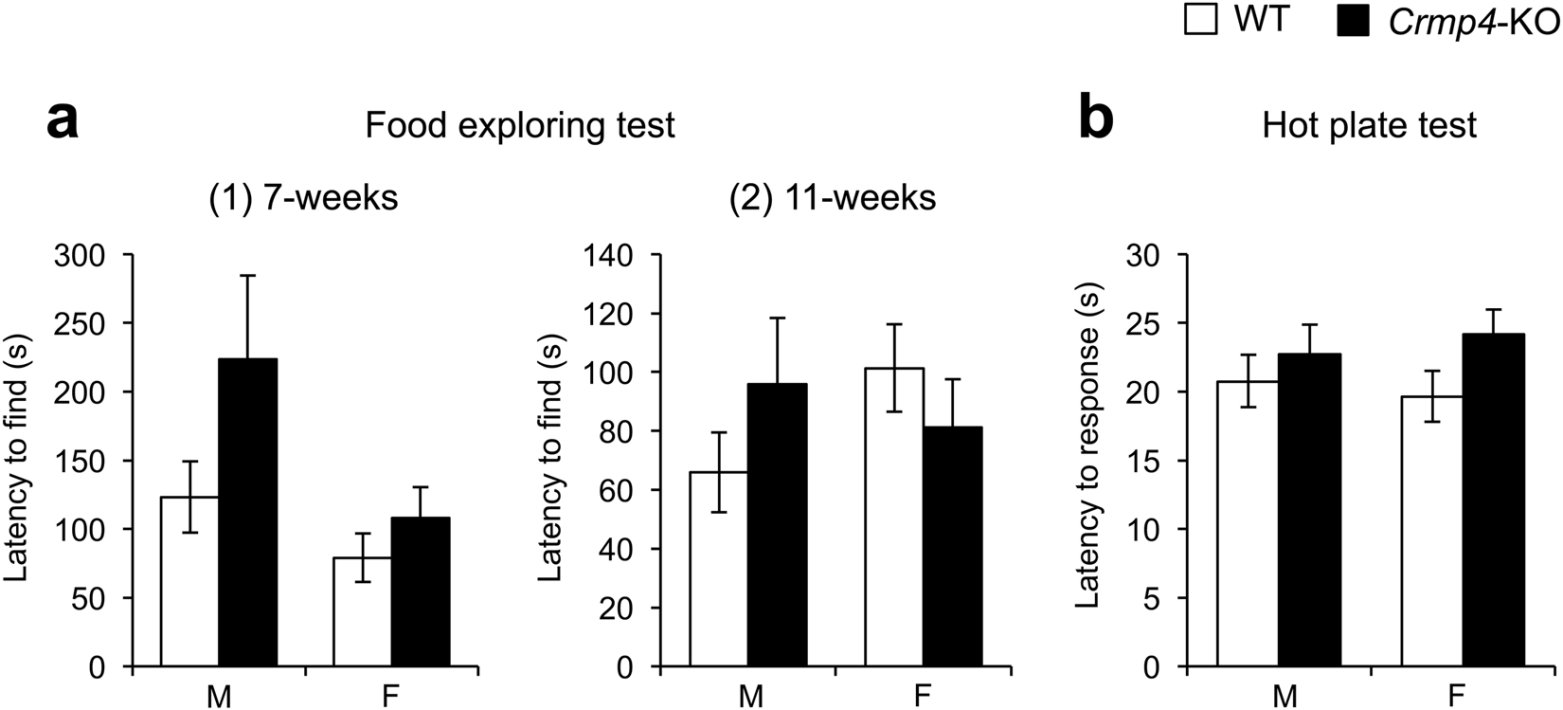
Food exploring and hot plate tests. (**a**) Food exploring test at 7-weeks [(1), male WT, n = 15; male *Crmp4*-KO, n = 15; female WT, n = 12; and female *Crmp4*-KO, n = 16] and 11-weeks [(2), male WT, n = 11; male *Crmp4*-KO, n = 15; female WT, n = 10; and female *Crmp4*-KO, n = 15] to measure time needed to find buried food. (**b**) Hot plate test to assess acute pain sensitivity to a thermal stimulus. The time needed to respond with a hind paw lick, paw shake or jump was measured. Male WT, n = 21; male *Crmp4*-KO, n = 16; female WT, n = 26; and female *Crmp4*-KO, n = 24. Values are expressed as mean ± SEM.

We also tested the olfactory function in mice using the food exploring test at 7 and 11 weeks of age because an olfactory deficit would interfere with social behaviour (Fig. 5a). Although *Crmp4-*KO males required more time to find food than mice in other test groups at 7 weeks of age (Fig. 5a-(1)), there were no significant differences, as determined by two-way ANOVA (Supplementary Table S1). The food exploring test in 11-week-old mice also revealed no significant differences in the olfactory function between genotypes and sexes (Fig. 5a-(2), Supplementary Table S1).

### *Crmp4*-KO mice show normal pain sensitivity

Next, to assess pain sensitivity, the hot plate test at 55 °C was performed at 8 weeks of age (Fig. 5b). The time required to respond to thermal stimulation did not differ significantly between WT and *Crmp4*-KO mice of either sex.

### Familiar and unfamiliar beddings induced different emission of ultrasonic vocalisations (UVs) in WT but not in *Crmp4*-KO pups

An association has been proposed between the patterns of sensory differences and core deficits of ASD^22^. Sensory differences are now included in the DSM-5 diagnostic criteria for ASD^23^. The emission of UVs by mouse pups is a social behaviour that shows attachment to the mother. In addition, UVs are the incidental by-product of normal physiological responses to sensory stimulation in neonatal mice^24^. The experiments of UVs were performed at PD7 because previous studies have shown that C57/BL6 mice pups produce more UVs at PD7 than at any other postnatal stage^25,26^.

**Figure 6:**
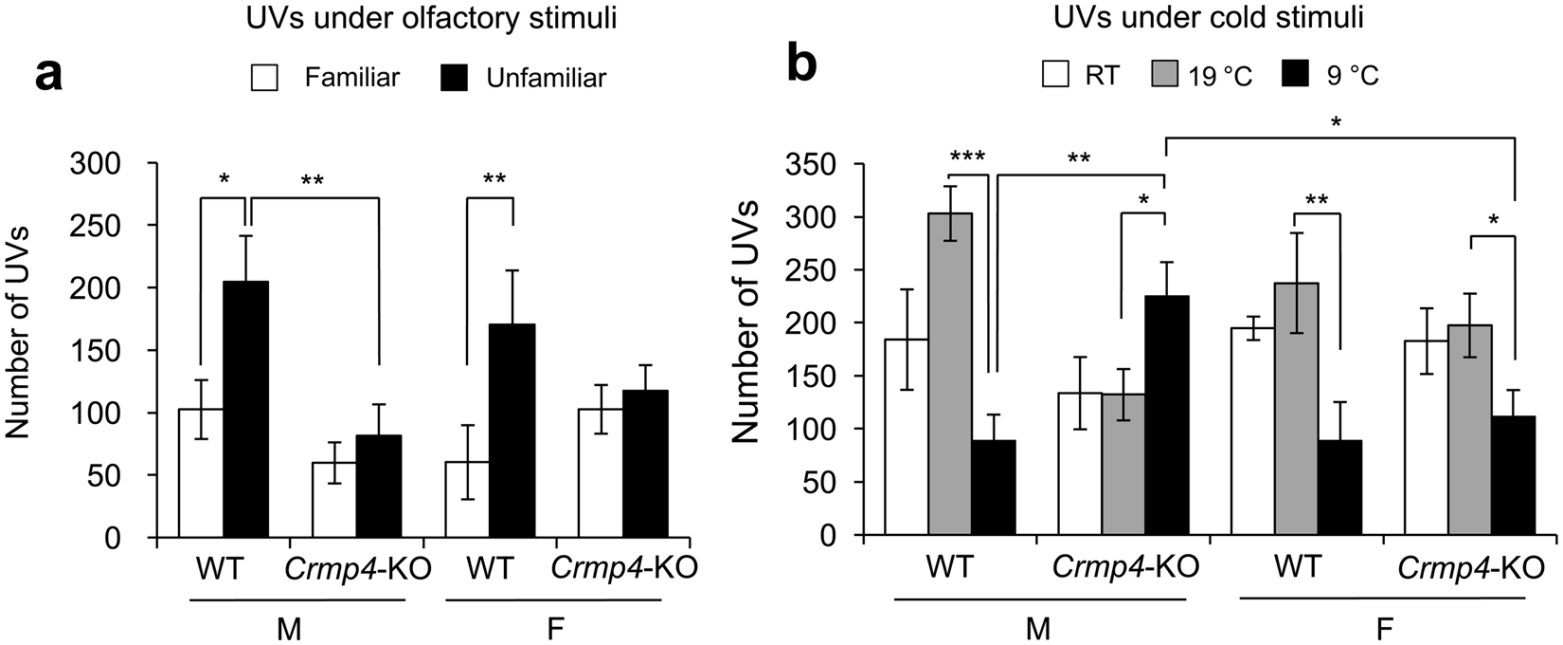
Ultrasonic vocalisations (UVs) emitted by mouse pups under different sensory stimuli. **(a)** Mean numbers of UVs emitted from wild-type [WT (male, n = 11: female, n = 12)] and *Crmp4*-knockout pups (male, n = 14: female, n = 16) were compared under exposure to familiar and unfamiliar nest bedding (**b**) and under exposure at 23 °C [room temperature, RT (male WT, n = 8; male *Crmp4*-KO, n = 8; female WT, n = 13; and female *Crmp4*-KO, n = 6)], 19 °C (male WT, n = 11; male *Crmp4*-KO, n = 15; female WT, n = 14; and female *Crmp4*-KO, n = 16), and 9 °C (male WT, n = 13; male *Crmp4*-KO, n = 14; female WT, n = 12; and female *Crmp4*-KO, n = 10). Values are expressed as mean ± SEM. *p < 0.05, **p < 0.01, ***p < 0.001 (three-way ANOVA followed by Fisher’s PLSD *post*-*hoc* tests).

The number of UVs emitted by pups under two different smell stimuli, familiar and unfamiliar nest bedding, was counted (Fig. 6a). Three-way ANOVA showed that exposure to unfamiliar bedding induced significantly more UVs than that to familiar bedding (*p* < 0.01) and that *Crmp4*-KO pups produced significantly less UVs than WT pups (*p* < 0.05). In addition, two significant interactions (genotype × bedding and genotype × sex, *p* < 0.05 each) were identified. The number of UVs produced by WT pups of both sexes was significantly greater during exposure to the unfamiliar bedding than during exposure to the familiar bedding (males and females, *p* < 0.05 and *p* < 0.01, respectively, Fig. 6a), as reported by previous studies^27–29^. In contrast, the number of UVs emitted by male and female *Crmp4*-KO pups did not differ significantly between familiar and unfamiliar bedding (Fig. 6a). These results suggest that while WT pups of both sexes could discriminate between the smell of familiar and unfamiliar bedding, *Crmp4*-KO pups of both sexes could not discriminate well between these two bedding types. It is also possible that the pups could retain this ability but did not change their behaviour. In addition, a significant difference in the number of UVs between WT and *Crmp4*-KO pups in response to unfamiliar bedding was shown in males but not in females (*p* < 0.01, Fig. 6a).

### Male *Crmp4*-KO pups showed significant abnormalities in thermal perception compared with WT pups

Because some human ASD patients are reported to have abnormalities in thermal detection^30,31^, we examined the thermal perception in *Crmp4*-KO mice using UV emissions at PD7 (Fig. 6b). Three-way ANOVA revealed that the number of UVs was significantly affected by temperature (*p* < 0.001, Supplementary table S1), and it also showed two significant interactions (genotype × temperature and genotype × sex × temperature). As shown in Fig. 6b, when male WT pups were kept at 9 °C, they produced significantly fewer UVs than those kept at 19 °C (*p* < 0.001). Similarly, the number of UVs was greater at 19 °C than at 9 °C in female WTs and *Crmp4-*KOs (*p* < 0.01 and *p* < 0.05, respectively). Surprisingly, in contrast to other animal groups, the number of UVs was greater at 9 °C than at 19 °C in *Crmp4-*KO males (*p* < 0.05).

In addition, no significant differences were observed in the number of UVs emitted at RT, 19 °C or 9 °C between female WT and *Crmp4*-KO pups. However, significant differences were found in the number of UVs between male WT and *Crmp4*-KO pups when they were kept at 9 °C (*p* < 0.01). The significant difference was also found in the number of UVs emitted at 9 °C between male and female *Crmp4*-KO pups (*p* < 0.05).

### Altered and sexually dimorphic expression of genes related to neural excitation and inhibition in *Crmp4*-KO mice

We examined the mRNA expression of *Crmp4* and several genes for receptors, synthetic enzymes for neurotransmitters and transporters, most of which have been implicated in relation to ASD, such as glutamate, γ-aminobutyric acid (GABA) and dopamine receptor genes^32–34^. The expression of serotonin transporter (*SERT*) mRNA was additionally examined in the raphe nucleus where it is reported to be associated with human ASD susceptibility^35^. In addition, we examined the mRNA expression of genes related to cell adhesion, such as *Ncam1* and *N-cadherin*, because they are involved in establishing neural connectivity^36^. The samples for real-time RT-PCR were collected from mice at 8 weeks of age, since abnormal social behaviors were detected at 6 and 10 weeks of age by three chamber test. The gene expressions with significant differences among animal groups are listed in Table 1, and *p*-values for multiple comparisons are shown in Supplementary Table S3. The gene expressions with no significant differences are listed in Supplementary Table S4.

**Table 1:** Gene expressions with significant differences among groups in adults.

Brain region	Genes	*F*-statistics, degree of freedom, and *p*-values for two-way ANOVA						Expression levels			
			For each factor and interaction			Fold differences		Male		Female	
			Genotype	Sex	Genotype × Sex	WT *vs*. *Crmp4*-KO (*Crmp4*-KO/WT)	Male *vs*. Female (Female/Male)	WT	*Crmp4*-KO	WT	*Crmp4*-KO
Olfactory bulb	*GluR2*	F(3,12) = 3.013, *p* = 0.072	F(1,12) = 5.843, *p* = 0.032	F(1,12) = 1.215, *p* = 0.292	F(1,12) = 1.981, *p = *0.185	1.969	1.352	0.532 ± 0.188	0.564 ± 0.141	0.472 ± 0.122	0.952 ± 0.120*
	*VGluT1*	F(3,12) = 16.871, *p* < 0.001	F(1,12) = 36.167, *p* < 0.001	F(1,12) = 11.327, *p* = 0.006	F(1,12) = 3.117, *p* = 0.103	1.389	0.833	0.815 ± 0.025^†^	1.009 ± 0.062*	0.585 ± 0.028	0.935 ± 0.053*
	*VGluT2*	F(3,12) = 3.191, *p* = 0.063	F(1,12) = 1.300, *p* = 0.276	F(1,12) = 5.707, *p* = 0.034	F(1,12) = 2.566, *p* = 0.135	1.116	1.266	0.849 ± 0.051	0.803 ± 0.029^†^	0.920 ± 0.170	1.172 ± 0.032
	*GABAAα1*	F(3,12) = 62.224, *p* < 0.001	F(1,12) = 78.549, *p* < 0.001	F(1,12) = 45.705, *p* < 0.001	F(1,12) = 62.418, *p* < 0.001	1.642	1.455	1.367 ± 0.120	1.459 ± 0.059^†^	1.259 ± 0.051	2.854 ± 0.126*
	*GABAAγ2*	F(3,12) = 11.147, *p* = 0.001	F(1,12) = 20.106, *p* = 0.001	F(1,12) = 1.439, *p* = 0.253	F(1,12) = 11.894, *p* = 0.005	1.524	1.118	1.274 ± 0.112	1.410 ± 0.194^†^	0.978 ± 0.059	2.021 ± 0.126*
	*GABABR1*	F(3,12) = 7.722, *p = *0.044	F(1,12) = 16.841, *p* = 0.001	F(1,12) = 4.138, *p* = 0.065	F(1,12) = 2.184, *p* = 0.165	1.839	1.342	0.182 ± 0.010	0.285 ± 0.068^†^	0.204 ± 0.036	0.424 ± 0.012*
	*VGAT*	F(3,12) = 7.897, *p = *0.004	F(1,12) = 12.939, *p* = 0.004	F(1,12) = 1.182, *p* = 0.298	F(1,12) = 9.570, *p* = 0.009	1.426	1.113	0.317 ± 0.029	0.335 ± 0.057^†^	0.249 ± 0.015	0.476 ± 0.018*
	*Ncam1*	F(3,12) = 7.576, *p = *0.004	F(1,12) = 20.543, *p* = 0.001	F(1,12) = 2.124, *p* = 0.166	F(1,12) = 0.012, *p* = 0.913	1.839	1.342	1.214 ± 0.043	1.573 ± 0.051*	1.085 ± 0.096	1.462 ± 0.113*
	*N-cadherin*	F(3,12) = 2.283, *p = *0.131	F(1,12) = 6.782, *p* = 0.023	F(1,12) = 0.007, *p* = 0.936	F(1,12) = 0.061, *p* = 0.809	1.321	0.913	1.178 ± 0.067	1.385 ± 0.041	1.149 ± 0.153	1.400 ± 0.035
Hippocampus	*GluR1*	F(3,12) = 3.793, *p* = 0.040	F(1,12) = 1.031, *p* = 0.330	F(1,12) = 0.916, *p* = 0.357	F(1,12) = 9.431, *p* = 0.010	1.171	0.861	0.544 ± 0.066	0.999 ± 0.121*^†^	0.860 ± 0.164	0.550 ± 0.114
	*GluR2*	F(3,12) = 2.857, *p* = 0.086	F(1,12) = 6.301, *p* = 0.029	F(1,12) = 0.106, *p* = 0.750	F(1,12) = 2.874, *p* = 0.118	1.505	1.055	0.227 ± 0.061	0.469 ± 0.044*	0.343 ± 0.022	0.400 ± 0.101
	*VGluT1*	F(3,12) = 1.942, *p* = 0.177	F(1,12) = 5.266, *p* = 0.041	F(1,12) = 0.169, *p* = 0.666	F(1,12) = 0.365, *p* = 0.557	1.160	0.973	0.916 ± 0.079	1.017 ± 0.042	0.850 ± 0.073	1.031 ± 0.047
	*GABAAα1*	F(3,12) = 3.623, *p* = 0.045	F(1,12) = 3.983, *p* = 0.069	F(1,12) = 5.771, *p* = 0.033	F(1,12) = 1.116, *p* = 0.312	1.211	1.265	0.194 ± 0.010	0.215 ± 0.010^†^	0.224 ± 0.010	0.291 ± 0.041
	*GABABR1*	F(3,12) = 3.548, *p* = 0.048	F(1,12) = 0.379, *p* = 0.549	F(1,12) = 10.185, *p* = 0.008	F(1,12) = 0.078, *p* = 0.784	1.071	1.514	0.135 ± 0.008	0.144 ± 0.008^†^	0.204 ± 0.034	0.220 ± 0.027
	*N-cadherin*	F(3,12) = 2.266, *p* = 0.133	F(1,12) = 5.333, *p* = 0.040	F(1,12) = 1.074, *p* = 0.321	F(1,12) = 0.392, *p* = 0.543	0.948	1.126	1.028 ± 0.046	0.939 ± 0.037	1.118 ± 0.032	1.096 ± 0.083
Cortex	*VGluT2*	F(3,12) = 3.920, *p* = 0.037	F(1,12) = 5.273, *p* = 0.040	F(1,12) = 6.351, *p* = 0.027	F(1,12) = 0.135, *p* = 0.719	0.735	0.712	0.136 ± 0.021	0.100 ± 0.009	0.097 ± 0.012	0.071 ± 0.007
	*GABAAγ2*	F(3,12) = 5.557, *p* = 0.013	F(1,12) = 14.229, *p* = 0.003	F(1,12) = 0.018, *p* = 0.896	F(1,12) = 2.425, *p* = 0.145	0.667	1.011	0.097 ± 0.013	0.077 ± 0.006	0.113 ± 0.011	0.064 ± 0.004*
	*GABABR1*	F(3,12) = 5.623, *p* = 0.012	F(1,12) = 7.181, *p = *0.020	F(1,12) = 7.752, *p* = 0.017	F(1,12) = 1.937, *p* = 0.189	0.773	0.765	0.115 ± 0.012^†^	0.081 ± 0.008*	0.080 ± 0.006	0.069 ± 0.007
	*Ncam1*	F(3,12) = 9.911, *p* = 0.001	F(1,12) = 29.650, *p < *0.001	F(1,12) = 0.011, *p* = 0.919	F(1,12) = 0.071, *p* = 0.795	0.671	1.008	1.227 ± 0.085	0.839 ± 0.068*	1.255 ± 0.084	0.827 ± 0.060*
	*N-cadherin*	F(3,12) = 20.827, *p* < 0.001	F(1,12) = 6.446, *p = *0.026	F(1,12) = 53.076, *p* < 0.001	F(1,12) = 2.960, *p* = 0.111	0.484	1.276	1.105 ± 0.121	0.592 ± 0.029*	1.498 ± 0.117	0.667 ± 0.070*

Asterisks indicate significant differences between WT and *Crmp4*-KO mice of same sex (*p < 0.05, two-way ANOVA followed by Fisher’s PLSD *post*-*hoc* tests). Dagger indicates significant difference between male and female mice of the same genotype (^†^ *p* < 0.05, two-way ANOVA followed by Fisher’s PLSD *post*-*hoc* tests). Data are expressed as mean ± SEM.

*Crmp4* mRNA expression was not detected in *Crmp4*-KO mice and it was not significantly different between male and female WT adults in the olfactory bulb (OB), hippocampus, and cortex (Supplementary Table S4). We found significant differences by genotype, sex, or interaction of these factors in *GluR1*, *GluR2*, *VGluT1*, *VGluT2*, *GABAAα1*, *GABAAγ2*, *GABABR1*, *VGAT*, *Ncam1* and *N-cadherin* mRNA expressions (two-way ANOVA) (Table 1). However, *Crmp4* deficiency-dependent effects in mRNA expression of these genes varied depending on the sex and brain region.

In the OB, *GluR2* mRNA expression in *Crmp4*-KO females was significantly higher than in WT females. *VgluT1* and *Ncam1* mRNA expressions in the OB were significantly higher in *Crmp4*-KO mice of both sexes than in WT mice of the same sex. In addition, *VgluT1* mRNA expression was significantly lower in WT females than in WT males. *VgluT2* mRNA expression was significantly higher in *Crmp4*-KO females than in *Crmp4*-KO males. *GABAAα1*, *GABAAγ2*, *GABABR1*, and *VGAT* mRNA expressions were significantly higher in *Crmp4*-KO females than in WT females and *Crmp4*-KO males. A significantly higher *N-cadherin* expression in the OB was observed in *Crmp4*-KO mice than in WTs, but *post*-*hoc* comparison did not show any significant differences among the groups.

In the hippocampus, *GluR1* mRNA expression was significantly higher in *Crmp4*-KO males than in WT males and *Crmp4*-KO females. *Crmp4*-KO males also showed higher *GluR2* mRNA expression than WT males. Two-way ANOVA revealed that *VgluT1* mRNA expression was significantly higher in *Crmp4*-KO mice than in WTs; however, *post-hoc* tests did not show significant differences among the groups. *GABAAα1* and *GABABR1* mRNA expressions were significantly higher in *Crmp4*-KO females than in *Crmp4*-KO males. *N-cadherin* mRNA expression was significantly lower in *Crmp4*-KO mice than in WT mice, but *post*-*hoc* tests did not show significant differences among the groups.

In the cortex, *VgluT2* mRNA expression was significantly lower in *Crmp4-*KO mice than in WT mice, but *post-hoc* tests did not show significant differences among the groups. *GABAAγ2* and *GABABR1* mRNA expressions were significantly lower in *Crmp4*-KO mice than in WT mice. *Post-hoc* tests revealed that *GABAAγ2* mRNA expression was significantly lower in *Crmp4*-KO females than in WT females and that of *GABABR1* was significantly lower in *Crmp4*-KO males than in WT males. *GABABR1* mRNA expression was significantly lower in WT females than in WT males. *Ncam1* and *N-cadherin* mRNA expressions were significantly lower in *Crmp4*-KO mice than in WT mice of the same sex. Gene expression levels of *SERT* in the raphe and serotonin receptors (*5HT1A*, *5HT2A*, *and 5HT7*) and dopamine receptors (*D1R* and *D2R*) in the OB, hippocampus, and cortex did not significantly differ between *Crmp4*-KO and WT mice of either sex (Supplementary Table S4).

## Discussion

The association of damaging single-nucleotide variants and small insertion/deletion variations has been identified in a subset of ASD patients using massively parallel DNA sequencing^37^. In addition, exome sequencing has verified the causative *de novo* variants in 6–16% of ASD^38–41^. These methods have revealed hundreds of rare genetic variants that cause various symptoms associated with ASD. However, the relationship between rare ASD-associated variants and the pathogenesis of ASD remains poorly understood. A previous comprehensive study has discovered a *de novo* missense variant in *CRMP4* in an ASD proband^12^. In the present study, we found a *de novo* damaging variant in *CRMP4* in a boy diagnosed with ASD, and we further revealed that the mutation induces altered CRMP4 function on dendritic branching. In addition, based on our finding of the human *CRMP4* mutation, we provide evidence that deficiency of the murine *Crmp4* gene and protein results in behavioural and sensory perceptual alterations related to human autistic behaviours in the animal. Surprisingly, most behavioural deficits in *Crmp4*-KO mice were more evident in males than in females. Moreover, compared with WT mice, *Crmp4*-KO mice showed significant changes in the expression of genes related to excitatory and inhibitory neurotransmission as well as in genes contributing to cell adhesion.

Decreased active interaction is a core behavioural deficit relevant to ASD^42^. In mouse models, the active interaction of the animal with a stranger is thought to be a core paradigm to test autistic behaviour^43^. The three-chamber and social interaction tests in our study indicated decreased social behaviour in male *Crmp4*-KO mice. In addition, the reduced emission of UVs from mouse pups on isolation to unfamiliar clean materials has been regarded as a behavioural alteration related to ASD^44,45^. We found that *Crmp4*-KO mice produced significantly decreased emission of UVs on exposure to unfamiliar clean bedding than WT mice. These results indicate that some behavioural characteristics relevant to ASD were observed in *Crmp4*-KO mice, some of which had a clearly male predominance.

Sensory disorders have also been reported to be considerably related to the core deficits of ASD^22^. In 2013, sensory differences, hyper- or hypo-reactivity, were added to the DSM-5 diagnostic criteria for ASD. In our study, we examined the responses of pups and adults to four kinds of sensory stimuli using the food exploring, hot plate and UV emission tests under olfactory (familiar and unfamiliar nest odours) and cold stimuli. The food exploring and hot plate tests showed that *Crmp4*-KO and WT adults have similar olfactory function and pain sensitivity. However, *Crmp4*-KO pups differently responded on exposure to olfactory and cold stimuli. *Crmp4-*KO pups, particularly males, could not discriminate well between familiar and unfamiliar beddings. In addition, abnormal thermal sensitivity was found at a low temperature (9 °C, Fig. 6b) in male *Crmp4*-KO pups.

The mechanism causing these behavioural and sensory alterations in *Crmp4*-KO mice, most of which had a male dominance, remains uncertain. However, our gene expression analysis may provide a clue. As shown in Table 1, *Crmp4* deficiency altered the mRNA expression of eight genes related to neurotransmission among those examined: *GluR1*, *GluR2*, *VgluT1*, *VgluT2*, *GABAα1*, *GABAAγ2*, *BAGABR1*, and *VGAT*. Previous studies have indicated the contributions of *GluR1* and *GluR2* in ASD^33,46,47^; these receptors are members of the AMPA-type receptor family that mediates fast excitatory transmission. VgluT1 and VgluT2 have an important role in glutamate transportation in the synaptic vesicle membrane. The mRNA expressions of glutamatergic neurotransmission related genes were increased in the OB and hippocampus in *Crmp4*-KO males, females, or both male and females than in WT mice of the same sex (Table 1). We also observed a female-specific increase in the mRNA expression of genes associated with inhibitory transmission, such as *GABAAα1*, *GABAAγ2*, *GABABR1*, and *VGAT* in the OB and of *GABAAα1* and *GABABR1* in the hippocampus of adult *Crmp4*-KO mice (Table 1). Increased glutamatergic neurotransmission can lead to an increased activity of excitatory neurons. In contrast, increased GABA neurotransmission would lead to suppression of neuronal activity. Therefore, in the OB and hippocampus of males, *Crmp4* deletion may possibly give rise to an increased ratio of excitation/inhibition, which has been proposed to be a key pathophysiological mechanism contributing to ASD^48,49^.

However, in the cortex, *GluR1* and *GluR2* mRNA expressions were not increased and *GABAAγ2* and *GABABR1* mRNA expressions were significantly decreased in *Crmp4*-KO mice than in WT mice. In addition, the number of genes with altered expressions due to *Crmp4* deletion differed among brain regions. These results suggest regionally different effects of *Crmp4* deletion on neurotransmission.

In contrast, although treatments targeting a specific molecular component of the glutamatergic system are likely to benefit only a subset of the ASD population, glutamatergic medication has been used in the treatment of ASD^50^, supporting the involvement of the glutamatergic system in ASD pathogenesis. However, Purcell *et al*. have reported that ASD patients exhibit elevated expression of GluR1^46^, while Ramanathan *et al*. found hemizygous deletion of *GluR2* gene in an ASD patient^51^. The role of GluRs in ASD pathogenesis still remains controversial. In some mouse models with targeted mutations in candidate genes for ASD, abnormal expression of *GluR1* and *GluR2* was also reported. Schmeisser *et al*. have demonstrated that genetic deletion of ProSAP1/Shank2, one of the famous mouse models of ASD, results in an early brain-region-specific upregulation of ionotropic glutamate receptors at the synapse^52^. A mouse model of Fragile X syndrome is sometimes used in studies of ASD. Pilpel *et al*. have reported that a mouse model of Fragile X syndrome (*Fmr1* KO2) displays a significantly lower AMPA to NMDA ratio than WTs at 2 weeks of post-natal development but not at 6–7 weeks of age^53^. Combining these data with our present data leads to a hypothesis that the time-, region- and gender-dependent changes in gene expressions associated with glutamatergic as well as GABAergic transmission induced by *Crmp4* deletion could be involved in processes underlying the pathogenesis of some features of human ASD.

CRMP4 is a member of the CRMP family of proteins (CRMP1–5) which are thought to regulate cell proliferation, migration, neuronal differentiation and signal transduction through their interaction with tubulin and actin^54–56^. Our previous proteomics study on the sexually dimorphic nucleus (AVPV) in the hypothalamic area, a region thought to be critical for generating the pre-ovulatory GnRH/LH surge in females, identified CRMP4 as a protein exhibiting sex-based differential expression during sexual differentiation of the nucleus^11^, although *Crmp4* function in the sexual differentiation of AVPV remains unclear. Some studies have shown that CRMP4 negatively regulates axonal growth^57–60^, while others have shown its positive regulation of axonal elongation^61,62^. In addition, CRMP4 is involved in dendrite formation^18,19,63^. In our study, we confirmed that CRMP4 suppresses the length of dendrites and number of dendritic branches (Fig. 2). Real-time qRT-PCR analysis revealed significant changes in *Ncam1* and *N-cadherin* mRNA expressions, two of the factors that play important roles in cell adhesion and dendritic growth^36^. Furthermore, CRMP4 is known to functionally interact with RhoA^58,61^, which has significant effects on axon elongation and spine morphogenesis^64^. NMDA-induced calpain activation leads to degradation of CRMP4^65^, which may contribute to the alteration of spine density^66^. A regulatory role of CRMP4 has been demonstrated during the migration of neuroblastoma cells through the binding with chondroitin sulfate in the extracellular matrix of the cortical plate^67^. Interestingly, S541 in *CRMP4*, which was mutated into Y541 in the *de novo* variant of our ASD patient, has been reported to be phosphorylated^68^. Because phosphorylation is critical for functions of CRMPs, the mutation in CRMP4 may impair its suppressive role in dendritic branching, as shown in Fig. 2.

On the other hand, *Crmp4* mRNA expression in the three brain regions tested were not significantly different between sexes. This is not surprising because our previous study found that the sex-differential expressions of CRMP4 and *Crmp4* mRNA was seen only when the sex differentiation occurs in the sexual dimorphic nucleus of the hypothalamus^11^. Since the expression of *Crmp4* mRNA in adult brain is very weak^69^, the different effects on gene expression and social behavior between male and female *Crmp4*-KO mice might be the result of the difference during neural network formation. Collectively, these observations indicate that CRMP4 plays important roles as one of the convergent regulators in neural network formation and that its dysfunction may lead to abnormalities in gene expression resulting in abnormal behaviours in mice. In addition, the regulatory role for CRMP4 in neural network formation are consistent with the hypothesis that CRMP4 disruption might contribute to ASD symptoms.

In summary, we found a damaging *de novo* missense *CRMP4* mutation in a male ASD patient. We also found that *Crmp4*-KO mice have abnormalities that are relevant to some phenotypes of human ASD. Accumulating evidence from previous and present studies on CRMP4 suggests that a disturbance in the regulatory function of CRMP4 in forming neuronal networks is an important aspect underlying some characteristics of ASD, including sexual differences. Our findings provide CRMP4 as a novel candidate gene contributing to ASD and shed new light on the role of CRMP4 in sex differences.

## Methods

### Case study of an ASD patient and whole-exome sequencing

Patient A379 and his parents were enrolled in the CORA following voluntary written informed consent as part of a human subjects research protocol approved by the Nationwide Children’s Hospital Institutional Review Board as required by 32 CFR 219 of the US Department of Health and Human Services and AFI 40-402 of the US Air Force. The patient (A379 in Fig. 1) is a 16-year-old Caucasian male diagnosed at age 5 years with an ASD. The history of the patient and method for exome sequencing are provided in Supplementary Information.

### Animals

WT and *Crmp4*-KO mice^18^ were generated by mating *Crmp4* ^+/−^ heterozygotes backcrossed onto the C57BL/6N background for at least 10 generations. Genotyping of the *Crmp4* allele was performed as previously described^18^. All the mice were weaned on PD21 and were housed in groups of 2–4 per cage, segregated by sex, in a room with a 12 h/12 h light–dark cycle (light on at 7:00 AM, off at 7:00 PM) and the temperature was maintained at 23 ± 1 °C. Food and water were available *ad libitum*. The plan for the use and care of the animals and methods were reviewed and approved by the Institutional Animal Care and Use and Ethical Committee of the University of Tokyo and Toyo University. All methods were performed in accordance with the relevant guidelines and regulations.

### Primary cultures of hippocampal cells from WT and *CRMP4*-KO mice and *CRMP4*-KO cells transfected with either *CRMP4* or *CRMP4*^S540Y^ expression vector

Primary culture was performed as previously reported^19^. In this study, to assess the general effects of *Crmp4* or *Crmp4* ^S540^ expressions on dendritic growth, only male animals were used for primary culture. In addition, we used hippocampal pyramidal neurons because they are routinely used to examine the effects on neurite outgrowth. *Crmp4* (pEGFP-*Crmp4* vector) and *Crmp4* ^S540Y^ (pEGFP-*Crmp4* ^S540Y^) expression vectors were constructed as described in Supplementary Information.

Either pEGFP-*Crmp4*, pEGFP-*Crmp4* ^S540Y^ or pEGFP-N (provided by H. Okado, Tokyo Metropolitan Institute of Medical Science) vector was transfected into the cultured hippocampal cells removed from *Crmp4*-KO mice using Lipofectamine 3000 (Invitrogen) on day *in vitro* 1 (DIV-1). Hippocampal cells were fixed on DIV-3 with 4% paraformaldehyde and then immunocytochemically stained as previously descried^19^. The total length of dendrites and number of branching points were estimated on MAP2-positive dendrites of each neuron with EGFP labelling.

### Behavioural experiments

All the behavioural experiments were performed from 1:00 PM to 5:00 PM in the behaviour-testing room. Mice at PD7 were tested for UVs. The cages containing the mice were transferred to the behaviour-testing room 30 min before commencement of the first trial.

### Open-field test

The open-field test was performed as previously described^70^, with minor modifications. Each mouse (male WT, n = 17; male *Crmp4*-KO, n = 15; female WT, n = 12; and female *Crmp4*-KO, n = 14) was placed in the corner of the open-field apparatus (50 × 50 × 32 cm, Muromachi Kikai Co., Ltd., Tokyo, Japan) facing the wall. Each subject was given a 20-min test. The total distance travelled (cm), velocity (cm/min) and time (s) spent in the centre (35 × 35 cm, inner square) were determined using the Noldus Ethovision system (Noldus, Leesburg, VA, USA).

### Elevated plus maze test

The elevated plus maze test was performed as previously described^71^, with minor modifications. Each mouse (male WT, n = 11; male *Crmp4*-KO, n = 10; female WT, n = 9; and female *Crmp4*-KO, n = 12) was tested on an elevated plus maze. Briefly, a mouse was placed on the central platform facing a close arm and allowed to freely traverse the maze for 10 min. The time spent in each arm and the number of arm entries (70% of mice in an arm) were measured.

### Novel object recognition test

The novel object recognition test was performed, as previously described^72^. Each mouse (male WT, n = 13; male *Crmp4*-KO, n = 17; female WT, n = 13; and female *Crmp4*-KO, n = 13) was individually habituated to the open-field box with 10 min of exploration in the absence of objects 24 h before starting the familiarisation phase. The protocols for the novel object recognition test and figures of the objects are provided in Supplementary Fig. S1 and its legend.

### Three-chamber test

The three-chamber test was performed as previously described^71^, with minor modifications. Each tested mouse at 6 weeks of age (male WT, n = 11; male *Crmp4*-KO, n = 10; female WT, n = 11; and female *Crmp4*-KO, n = 12) or at 10 weeks of age (male WT, n = 10; male *Crmp4*-KO, n = 8; female WT, n = 9; and female *Crmp4*-KO, n = 11) was individually placed in the centre chamber and allowed to freely explore all three chambers for 10 min while movement was video-recorded. The time spent in each chamber and that spent sniffing were measured.

### Social interaction test

The social interaction test was performed, as previously described^73^. Each mouse (male WT, n = 15; male *Crmp4*-KO, n = 17; female WT, n = 12; and female *Crmp4*-KO, n = 17) was maintained in social isolation for 1 week by placing one mouse/cage under the same conditions as described above. On the experimental day, after a 10-min habituation to the sound-attenuating chamber, an age-matched, unfamiliar C57BL/6N WT mouse of the same sex and similar body weight (within 5%) was placed in the corner of the cage. The duration of active interaction behaviours such as sniffing, mounting and chasing was measured.

### Tube test for social dominance

Thirty WT and *Crmp4*-KO mice (six males and nine females in each group) were tested, as previously described^73^. Each pairing was performed twice for a total of 30 matches. The winning percentage was calculated for each group.

### Food exploring test

The food exploring test was performed, as previously described^74^, with minor modifications. Each mouse at 7 weeks of age (male WT, n = 15; male *Crmp4*-KO, n = 15; female WT, n = 12; and female *Crmp4*-KO, n = 16) or at 11 weeks of age (male WT, n = 11; male *Crmp4*-KO, n = 15; female WT, n = 10; and female *Crmp4*-KO, n = 15) was deprived of food for 18 h before testing, while water was freely available. A piece of a food pellet (0.5 g) was buried beneath clean bedding of about 0.5 cm at the centre of the cage. A mouse was placed in the corner of the cage and the time required to find the food was determined.

### Hot plate test

The hot plate test was performed, as previously described^75^. Each mouse (male WT, n = 21; male *Crmp4*-KO, n = 16; female WT, n = 26; and female *Crmp4*-KO, n = 24) was placed on a heating apparatus maintained at 55 °C. The time until the first hind-paw response was determined. The paw response was defined as a paw lick, a foot shake or jumping. A cutoff time of 60 s was used.

### Measurement of UVs emitted by mouse pups under different sensory stimuli

UV assessment of WT (male, n = 11; female, n = 12) and *Crmp4*-KO (male, n = 14; female, n = 16) littermates at PD7 was performed under familiar or unfamiliar odour, as previously reported^27^.

Next, we examined UVs emitted by WT and *Crmp4*-KO pups in response to thermal change. We used WT and *Crmp4*-KO pups of both sexes and isolated them at RT, 19 °C or 9 °C. Each pup (male WT, n = 8; female WT, n = 13; male *Crmp4*-KO, n = 8; female *Crmp4*-KO, n = 6) was isolated from the nest in a clean plastic case at RT. Other pups (WT male, n = 24; WT female, n = 26; male *Crmp4*-KO, n = 29; female *Crmp4*-KO, n = 26) were isolated from the nest in a clean glass beaker maintained at 19 °C or 9 °C. In each test, UVs were recorded for 5 min.

### Real-time qRT-PCR

Complete description is provided in Supplementary Information.

### Statistical analysis

Data were analysed using the SPSS 22.0 software (SPSS Inc., Chicago, IL, USA). For statistical comparisons, one-, two- or three-way ANOVA followed by Fischer’s protected least significant difference (PLSD) test, Bonferroni correction, Sidak correction, and Tukey HSD test were conducted. The binomial test was used for the tube test. In all analyses, *p* < 0.05 was regarded as statistically significant. For direct pairwise comparisons, Student’s *t-*test was applied.

### Data Availability

All data generated or analysed during this study are included in this published article (and its Supplementary Information files).

## Electronic supplementary material

Supplementary Information

## References

[1] Kanner L (1943). Autistic disturbances of affective contact. Nervous Child.

[2] Fombonne E (2003). Epidemiological surveys of autism and other pervasive developmental disorders: An update. J Autism Dev Disord.

[3] Christensen, D. L. *et al*. Prevalence and Characteristics of Autism Spectrum Disorder Among Children Aged 8 Years - Autism and Developmental Disabilities Monitoring Network, 11 Sites, United States, 2012. *MMWR Surveill Summ* **65**, 10.15585/mmwr.ss6503a1 (2016). 10.15585/mmwr.ss6503a1 PMC7909709 27031587

[4] O’Roak BJ (2014). Recurrent de novo mutations implicate novel genes underlying simplex autism risk. Nat Commun.

[5] Sato D (2012). SHANK1 Deletions in Males with Autism Spectrum Disorder. Am J Hum Genet.

[6] Hu VW, Sarachana T, Sherrard RM, Kocher KM (2015). Investigation of sex differences in the expression of RORA and its transcriptional targets in the brain as a potential contributor to the sex bias in autism. Mol Autism.

[7] Kim KC (2016). MeCP2 Modulates Sex Differences in the Postsynaptic Development of the Valproate Animal Model of Autism. Mol Neurobiol.

[8] Baron-Cohen S (2011). Why are autism spectrum conditions more prevalent in males?. PLoS Biol.

[9] Blumberg SJ (2013). Changes in prevalence of parent-reported autism spectrum disorder in school-aged U. S. children: 2007 to 2011–2012. Natl Health Stat Report.

[10] Idring S (2015). Changes in prevalence of autism spectrum disorders in 2001–2011: findings from the Stockholm youth cohort. J Autism Dev Disord.

[11] Iwakura T (2013). Collapsin response mediator protein 4 affects the number of tyrosine hydroxylase-immunoreactive neurons in the sexually dimorphic nucleus in female mice. Dev Neurobiol.

[12] Iossifov I (2014). The contribution of de novo coding mutations to autism spectrum disorder. Nature.

[13] Smith RM, Banks W, Hansen E, Sadee W, Herman GE (2014). Family-based clinical associations and functional characterization of the serotonin 2A receptor gene (HTR2A) in autism spectrum disorder. Autism Res.

[14] Davydov EV (2010). Identifying a high fraction of the human genome to be under selective constraint using GERP++. PLoS Comput Biol.

[15] Kumar P, Henikoff S, Ng PC (2009). Predicting the effects of coding non-synonymous variants on protein function using the SIFT algorithm. Nat Protoc.

[16] Adzhubei IA (2010). A method and server for predicting damaging missense mutations. Nat Methods.

[17] Dong C (2015). Comparison and integration of deleteriousness prediction methods for nonsynonymous SNVs in whole exome sequencing studies. Hum Mol Genet.

[18] Niisato E (2012). *CRMP4* suppresses apical dendrite bifurcation of CA1 pyramidal neurons in the mouse hippocampus. Dev Neurobiol.

[19] Tsutiya A (2016). Deletion of collapsin response mediator protein 4 results in abnormal layer thickness and elongation of mitral cell apical dendrites in the neonatal olfactory bulb. J Anat.

[20] Montani C (2017). The X-Linked Intellectual Disability Protein IL1RAPL1 Regulates Dendrite Complexity. J Neurosci.

[21] Cheng N, Alshammari F, Hughes E, Khanbabaei M, Rho JM (2017). Dendritic overgrowth and elevated ERK signaling during neonatal development in a mouse model of autism. PLoS One.

[22] Boyd BA (2010). Sensory features and repetitive behaviors in children with autism and developmental delays. Autism Res.

[23] American Psychiatric Association. Diagnostic and Statistical Manual of Mental Disorders, 5th ed. American Psychiatric Association. 10.1176/appi.books.9780890425596.dsm01. (2013).

[24] Scattoni ML, Crawley J, Ricceri L (2009). Ultrasonic vocalizations: a tool for behavioural phenotyping of mouse models of neurodevelopmental disorders. Neurosci Biobehav Rev.

[25] Fish EW, Sekinda M, Ferrari PF, Dirks A, Miczek KA (2000). Distress vocalizations in maternally separated mouse pups: modulation via 5-HT(1A), 5-HT(1B) and GABA(A) receptors. Psychopharmacology.

[26] Lemasson M, Delbé C, Gheusi G, Vincent JD, Lledo PM (2005). Use of ultrasonic vocalizations to assess olfactory detection in mouse pups treated with 3-methylindole. Behav Process.

[27] Tsutiya A, Nishihara M, Goshima Y, Ohtani-Kaneko R (2015). Mouse pups lacking collapsin response mediator protein 4 (*CRMP4*) manifest impaired olfactory function and hyperactivity in the olfactory bulb. Eur J Neurosci.

[28] Moles A, Kieffer BL, D’Amato FR (2004). Deficit in attachment behavior in mice lacking the mu-opioid receptor gene. Science.

[29] Wöhr M (2015). Effect of social odor context on the emission of isolation-induced ultrasonic vocalizations in the BTBR T+tf/J mouse model for autism. Front Neurosci.

[30] Duerden EG (2015). Decreased sensitivity to thermal stimuli in adolescents with autism spectrum disorder: relation to symptomatology and cognitive ability. J Pain.

[31] Yasuda Y (2016). Sensory cognitive abnormalities of pain in autism spectrum disorder: a case-control study. Ann Gen Psychiatry.

[32] de Krom M (2009). A common variant in DRD3 receptor is associated with autism spectrum disorder. Biol Psychiatry.

[33] Uzunova G, Hollander E, Shepherd J (2014). The role of ionotropic glutamate receptors in childhood neurodevelopmental disorders: autism spectrum disorders and fragile x syndrome. Curr Neuropharmacol.

[34] Pizzarelli R, Cherubini E (2011). Alterations of GABAergic signaling in autism spectrum disorders. Neural Plast.

[35] Veenstra-VanderWeele J (2012). Autism gene variant causes hyperserotonemia, serotonin receptor hypersensitivity, social impairment and repetitive behavior. Proc Natl Acad Sci USA.

[36] Hansen SM, Berezin V, Bock E (2008). Signaling mechanisms of neurite outgrowth induced by the cell adhesion molecules NCAM and N-cadherin. Cell Mol Life Sci.

[37] Dong S (2014). De novo insertions and deletions of predominantly paternal origin are associated with autism spectrum disorder. Cell Rep.

[38] Iossifov I (2012). De novo Gene disruptions in children on the autistic spectrum. Neuron.

[39] Neale BM (2012). Patterns and rates of exonic de novo mutations in autism spectrum disorders. Nature.

[40] O’Roak BJ (2012). Sporadic autism exomes reveal a highly interconnected protein network of de novo mutations. Nature.

[41] Sanders SJ (2012). De novo mutations revealed by whole-exome sequencing are strongly associated with autism. Nature.

[42] Happe F, Ronald A (2008). The ‘fractionable autism triad’: a review of evidence from behavioural, genetic, cognitive and neural research. Neuropsychol Rev.

[43] Silverman JL, Yang M, Lord C, Crawley JN (2010). Behavioural phenotyping assays for mouse models of autism. Nat Rev Neurosci.

[44] Mosienko V, Beis D, Alenina N, Wöhr M (2015). Reduced isolation-induced pup ultrasonic communication in mouse pups lacking brain serotonin. Mol Autism.

[45] Wöhr M, Roullet FI, Hung AY, Sheng M, Crawley JN (2011). Communication impairments in mice lacking Shank1: reduced levels of ultrasonic vocalizations and scent marking behavior. PLoS One.

[46] Purcell AE, Jeon OH, Zimmerman AW, Blue ME, Pevsner J (2001). Postmortem brain abnormalities of the glutamate neurotransmitter system in autism. Neurology.

[47] Essa MM, Braidy N, Vijayan KR, Subash S, Guillemin GJ (2013). Excitotoxicity in the pathogenesis of autism. Neurotox Res.

[48] Gatto CL, Broadie K (2010). Genetic controls balancing excitatory and inhibitory synaptogenesis in neurodevelopmental disorder models. Front Synaptic Neurosci.

[49] Rubenstein JL (2010). Three hypotheses for developmental defects that may underlie some forms of autism spectrum disorder. Curr Opin Neurol.

[50] Fung LK, Hardan AY (2015). Developing Medications Targeting Glutamatergic Dysfunction in Autism: Progress to Date. CNS Drugs.

[51] Ramanathan S (2004). A case of autism with an interstitial deletion on 4q leading to hemizygosity for genes encoding for glutamine and glycine neurotransmitter receptor subunits (AMPA2, GLRA3, GLRB) and neuropeptide receptors NPY1R, NPY5R. BMC Med Genet.

[52] Schmeisser MJ (2012). Autistic-like behaviours and hyperactivity in mice lacking ProSAP1/Shank2. Nature.

[53] Pilpel Y (2009). Synaptic ionotropic glutamate receptors and plasticity are developmentally altered in the CA1 field of Fmr1 knockout mice. J Physiol.

[54] Fukata Y (2002). CRMP-2 binds to tubulin heterodimers to promote microtubule assembly. Nat Cell Biol.

[55] Charrier E (2003). Collapsin response mediator proteins (CRMPs): involvement in nervous system development and adult neurodegenerative disorders. Mol Neurobiol..

[56] Rosslenbroich V (2005). Collapsin response mediator protein-4 regulates F-actin bundling. Exp Cell Res.

[57] Alabed YZ, Pool M, Ong Tone S, Fournier AE (2007). Identification of *CRMP4* as a convergent regulator of axon outgrowth inhibition. J Neurosci.

[58] Alabed YZ, Pool M, Ong Tone S, Sutherland C, Fournier AE (2010). GSK3 beta regulates myelin-dependent axon outgrowth inhibition through *CRMP4*. J Neurosci.

[59] Nagai J (2015). *Crmp4* deletion promotes recovery from spinal cord injury by neuroprotection and limited scar formation. Sci Rep.

[60] Nagai J, Takaya R, Piao W, Goshima Y, Ohshima T (2016). Deletion of *Crmp4* attenuates CSPG-induced inhibition of axonal growth and induces nociceptive recovery after spinal cord injury. Mol Cell Neurosci.

[61] Khazaei MR (2014). Collapsin response mediator protein 4 regulates growth cone dynamics through the actin and microtubule cytoskeleton. J Biol Chem.

[62] Tan M (2015). *CRMP4* and CRMP2 Interact to Coordinate Cytoskeleton Dynamics, Regulating Growth Cone Development and Axon Elongation. Neural Plast.

[63] Cha C (2016). *CRMP4* regulates dendritic growth and maturation via the interaction with actin cytoskeleton in cultured hippocampal neurons. Brain Res Bull.

[64] Woolfrey KM, Srivastava DP (2016). Control of Dendritic Spine Morphological and Functional Plasticity by Small GTPases. Neural Plast.

[65] Kowara R, Ménard M, Brown L, Chakravathy B (2007). Co-localization and interaction of DPYSL3 and GAP43 in primary cortical neurons. Biochem Biophys Res Commun.

[66] Andres AL (2013). NMDA receptor activation and calpain contribute to disruption of dendritic spines by the stress neuropeptide CRH. J Neurosci.

[67] Franken S (2003). Collapsin response mediator proteins of neonatal rat brain interact with chondroitin sulfate. J Biol Chem.

[68] Mertins P (2016). Proteogenomics connects somatic mutations to signalling in breast cancer. Nature.

[69] Tsutiya A, Ohtani-Kaneko R (2012). Postnatal alteration of collapsin response mediator protein 4 mRNA expression in the mouse brain. J Anat..

[70] Umemori J (2013). ENU-mutagenesis mice with a non-synonymous mutation in Grin1 exhibit abnormal anxiety-like behaviors, impaired fear memory, and decreased acoustic startle response. BMC Res Notes.

[71] Weidner KL, Buenaventura DF, Chadman KK (2014). Mice over-expressing BDNF in forebrain neurons develop an altered behavioral phenotype with age. Behav Brain Res.

[72] Isono T (2013). Amyloid-β25–35 induces impairment of cognitive function and long-term potentiation through phosphorylation of collapsin response mediator protein 2. Neurosci Recearch.

[73] Sato A (2012). Rapamycin reverses impaired social interaction in mouse models of tuberous sclerosis complex. Nat Commun.

[74] Mitsui S (2009). A mental retardation gene, motopsin/neurotrypsin/prss12, modulates hippocampal function and social interaction. Eur J Neurosci.

[75] Hikida T, Kitabatake Y, Pastan I, Nakanishi S (2003). Acetylcholine enhancement in the nucleus accumbens prevents addictive behaviors of cocaine and morphine. Proc Natl Acad Sci USA.

