# Supplementary material for: Human CRMP4 mutation and disrupted Crmp4 expression in mice are associated with ASD characteristics and sexual dimorphism
Source: Sci Rep. 2017 Dec 1;7:16812. doi: 10.1038/s41598-017-16782-8 (PMC5711804; doi:10.1038/s41598-017-16782-8)
Supplement: Supplementary file 1 — Supplementary Information [file 41598_2017_16782_MOESM1_ESM.pdf]

Human *CRMP4* mutation and disrupted *Crmp4* expression in mice are associated with ASD characteristics and sexual dimorphism

Atsuhiro Tsutiya[1,6], Yui Nakano[2], Emily Hansen-Kiss[3], Benjamin Kelly[3], Masugi Nishihara[4], Yoshio Goshima[5], Don Corsmeier[3], Peter White[3], Gail E. Herman[3] and Ritsuko Ohtani-Kaneko[1,2,7*]

[1]Institute of Life Innovation Studies, Toyo University, 1-1-1 Itakura, Oura, Gunma 374-0193, Japan.

[2]Graduate School of Life Sciences, Toyo University, 1-1-1 Itakura, Oura, Gunma 374-0193, Japan.

[3]The Institute for Genomic Medicine, Nationwide Children's Hospital and Department of Pediatrics, The Ohio State University, Columbus, OH 43205, USA.

[4]Department of Veterinary Physiology, Graduate School of Agricultural and Life Sciences, The University of Tokyo, 1-1-1 Yayoi, Bunkyo-ku, Tokyo 113-8657, Japan.

[5]Department of Molecular Pharmacology and Neurobiology, Yokohama City University Graduate School of Medicine, 3-9 Fuku-ura, Kanazawa Ward, Yokohama 236-0004, Japan.

[6]Clinical Proteomics and Molecular Medicine, St. Marianna University Graduate School of Medicine, Kawasaki, Japan.

[7]Research Center for Biomedical Engineering, Toyo University, 2100 Kujirai, Kawagoe, Saitama 350-8585, Japan.

Supplementary Information

History of the ASD patient

The patient weighed 3153 g at birth after a 39-week gestation and was born to a 28-year-old woman, gravida 2, para 1–2, with one previous miscarriage. This pregnancy was achieved by administration of Clomid and was complicated by maternal tobacco use, as well as weight loss of 12 lbs. Delivery was vaginal with vacuum assistance, and the baby was discharged home with his mother.

The family history is unremarkable. The patient has an older and a younger brother, both of whom are healthy with normal development, as are both parents. There is no known consanguinity. The patient had normal development until 3 years of age, during which there was a loss of language and social skills. He also developed unusual behaviours, including unusual obsessions and repetitive tapping of objects and making clicking noises. He attended a special needs preschool and repeated first grade. He has an individual education programme, receives speech therapy, and has been treated with Ritalin for attention-deficit/hyperactivity disorder.

At 8 years of age, the patient was assessed by an experienced psychologist using the Autism Diagnostic Observation Scale (ADOS) and received a score of 13 (Module 3), consistent with the diagnosis of ASD. The Autism Diagnostic Interview-Revised (ADI-R),) assessment, performed at 10 years of age, was also consistent with the ASD diagnosis. At 12 years of age, the patient received a full scale IQ standard score of 68 on the Stanford-Binet Intelligence Scale as part of CORA, falling into the 'mildly impaired' range (non-verbal IQ score, 82 and verbal IQ score, 57).

Previous genetic testing revealed a normal 46, XY male karyotype and normal results in DNA testing for Fragile X syndrome. As part of CORA, an oligonucleotide microarray was

performed (NimbleGen CGX-3) in 2011 that also showed no abnormalities. He underwent exome sequencing in the NCH Biomedical Genomics Core (BGC).

Whole-exome sequencing

Exome sequencing was performed on 72 simplex trios (affected and both parents). A total of 69 *de novo* damaging variants were confirmed, of which seven were considered pathogenic or likely pathogenic based on the predicted change in the protein and reports in the literature demonstrating that the gene is involved in human ASD and/or intellectual disability (G. Herman, personal communication).

DNA samples for exome sequencing were prepared from lymphoblastoid cell lines and were processed using the Agilent SureSelectXT Target Enrichment System for the Illumina Paired End Sequencing Protocol (Agilent Technologies, Santa Clara, CA, USA). DNA libraries prepared from the patient and both parents were captured using SureSelect Human All Exon v4 probes. Paired-end 100 base-pair reads were generated for exome-enriched libraries sequenced on the Illumina HiSeq 2000 (Illumina, San Diego, CA, USA) to an average depth of 74×. Secondary analysis was performed using Churchill, a pipeline at NCH, for the discovery of human genetic variation[1]. A *de novo* variant of *CRMP4* found in the sequenced sample from the proband but not in the parents or siblings was confirmed by Sanger sequencing using DNA prepared directly from a sample of whole blood to avoid any possible cell-line artefacts. PCR was performed for exon 11 using the primers 5′-CAGGTCATGTTCCCCTGTCT-3′ and 5′-ACGCTGCCAAGATCTTCAAC-3′ to amplify a 402-bp fragment.

Crmp4 (pEGFP-Crmp4 vector) and Crmp4^{S540Y} (pEGFP- Crmp4^{S540Y}) expression vectors

For the pEGFP-*Crmp4* vector, a cDNA fragment containing the full-length coding region of the mouse *Crmp4* gene was inserted into the *Eco*RI and *Xho*I sites of the pEGFP-N vector (Clontech Laboratories, Inc., Mountain View, CA, USA). *Crmp4*^{S540Y} was made using the PrimSTAR mutagenesis kit (Takara Bio, Japan) according to the manufacturer's protocol using the WT *Crmp4* sequence as a template. The S540 in the mouse *Crmp4* is the homologous site of S541 in the human *CRMP4* sequence (NP_001278384.1). The following primers were used to amplify the mutant mouse sequence: forward, 5′- CACCAGTATGTTGCCGAATACAACATC -3′; reverse, 5′- GGCAACATACTGGTGGTTCTTGGCAGA -3′, where the underlined base results in the missense variant. Both constructs were sequenced on an ABI PRISM 310 Genetic Analyzer (Perkin Elmer Cetus), and both confirmed the WT and mutant sequences with no additional base changes.

Real-time qRT-PCR

WT and *Crmp4*-KO mice of both sexes at 8 weeks of age (n = 4 each) were deeply anesthetised with pentobarbital (50 mg/kg, i.p.), and their brains were removed from the skull. The OB, cortex, hippocampus and raphe were dissected out. RNA extraction, reverse transcription, and real-time qRT-PCR were performed as previously reported[2]. The primer pairs used are listed in Supplementary Table S3.

Supplementary References

1. Kelly, B. J. *et al*. Churchill: an ultra-fast, deterministic, highly scalable and balanced parallelization strategy for the discovery of human genetic variation in clinical and population-scale genomics. *Genome Biol* **16**, 6 (2015).
2. Tsutiya A & Ohtani-Kaneko R. Postnatal alteration of collapsin response mediator protein 4 mRNA expression in the mouse brain. *J Anat.* **221**, 341–351 (2012).

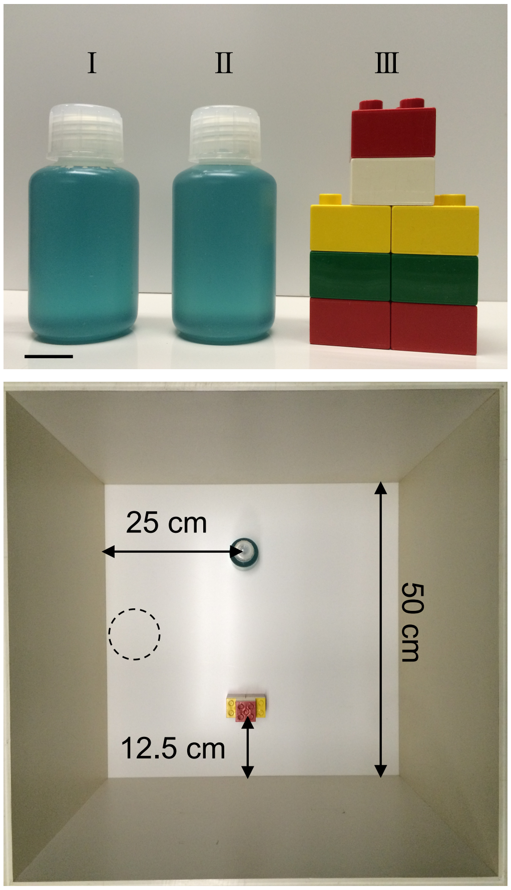

Supplementary Fig. S1. Novel object recognition test. During the familiarisation phase, two identical objects (‘I’ and ‘II’ in top panel) were located 12.5 cm away from the wall at the central width of the box (bottom panel). The mouse was then placed into the box, always facing the wall at the same position (dotted circle area in bottom panel) and allowed to freely explore for 10 min. Exploration of an object was defined as directing the nose to the object at a distance of less than 2 cm and/or touching it with the nose. After the test, the mouse was housed again. Object b was then replaced with a novel object (‘III’ in top panel), and the test phase was performed 60 min after the habituation phase. Each animal was placed at the same location (dotted circle area in bottom panel) and allowed to explore freely for 5 min. The total time spent exploring the two objects was measured during both the familiarisation and test phases. The preference index is the ratio of the amount of time spent exploring any one of the two objects (familiarisation phase) or the novel object (test phase) divided by the total time spent exploring both objects: [II/(I + II)] × 100 in the familiarisation phase and [III/(I + III)] × 100 in the test phase.

Supplementary Table S1.

Statistical values from ANOVA for behavioural analysis. Significant results are indicated in bold.

Test			Statistical method	*F*-statistics, degree of freedom, and *p*-values for ANOVA	*F*-statistics, degree of freedom, and *p*-values for ANOVA (for each effect and interaction)						
					Genotype			Sex			Genotype × Sex
Open field	Distance		Two-way ANOVA	F(3,54) = 0.805, *p* = 0.497	F(1,54) = 0.472, *p* = 0.495			F(1,54) = 0.434, *p* = 0.513			F(1,54) = 1.269, *p* = 0.265
	In zone center			F(3,54) = 1.066, *p* = 0.371	F(1.54) = 1.110, *p* = 0.297			F(1.54) = 1.082, *p* = 0.303			F(1.54) = 1.027, *p* = 0.315
	Velocity			F(3,54) = 0.788, *p* = 0.506	F(1,54) = 0.479, *p* = 0.492			F(1.54) = 0.393, *p* = 0.533			F(1.54) = 1.255, *p* = 0.268
Elevated plus maze	Time in open arms		Two-way ANOVA	F(3,39) = 0.625, *p* = 0.603	F(1,39) = 1.122, *p* = 0.296			F(1,39) = 0.308, *p* = 0.582			F(1,39) = 0.529, *p* = 0.471
	Time in closed arms			F(3,39) = 0.204, *p* = 0.893	F(1,39) = 0.159, *p* = 0.692			F(1,39) = 0.444, *p* = 0.509			F(1,39) = 0.043, *p* = 0.837
	No. of entries to open arms			F(3,39) = 0.208, *p* = 0.891	F(1,39) = 0.013, *p* = 0.909			F(1,39) = 0.001, *p* = 0.979			F(1,39) = 0.606, *p* = 0.441
	No. of entries to closed arms			F(3,39) = 1.736, *p* = 0.175	F(1,39) = 1.787, *p* = 0.190			F(1,39) = 3.967, *p* = 0.053			F(1,39) = 0.012, *p* = 0.912
Novel object recognition	Familiarisation	Preference	Two-way ANOVA	F(3,52) = 0.697, *p* = 0.558	F(1,52) = 0.801, *p* = 0.372			F(1,52) = 0.616, *p* = 0.436			F(1,52) = 0.762, *p* = 0.387
		Total time		F(3,52) = 0.065, *p* = 0.978	F(1,52) = 0.028, *p* = 0.868			F(1,52) = 0.038, *p* = 0.847			F(1,52) = 0.140, *p* = 0.709
	Test	Preference	Two-way ANOVA	F(3,52) = 0.075, *p* = 0.973	F(1,52) = 0.014, *p* = 0.906			F(1,52) = 0.077, *p* = 0.782			F(1,52) = 0.143, *p* = 0.707
		Total time		F(3,52) = 0.877, *p* = 0.459	F(1,52) = 1.172, *p* = 0.284			F(1,52) = 0.943, *p* = 0.336			F(1,52) = 0.224, *p* = 0.638
Three-chamber test					Genotype	Sex	Chamber	Genotype × Sex	Genotype × Chamber	Sex × Chamber	Genotype × Sex × Chamber
	6W	Duration (s)	Three-way ANOVA	**F(11,120) = 15.829, *p* < 0.001**	F(1,120) = 0.000, *p* = 1.000	F(1,120) = 0.000, *p* = 1.000	**F(2,120) = 81.648, *p* < 0.001**	F(1,120) = 0.000, *p* = 1.000	F(2,120) = 1.484, *p* = 0.231	F(2,120) = 0.902, *p* = 0.409	F(2,120) = 2.078, *p* = 0.130
		No. of entries		F(7,80) = 0.334, *p* = 0.936	F(1,80) = 1.083, *p* = 0.301	F(1,80) = 0.523, *p* = 0.472	F(1,80) = 0.002, *p* = 0.961	F(1,80) = 0.452, *p* = 0.503	F(1,80) = 0.260, *p* = 0.611	F(1,80) = 0.102, *p* = 0.750	F(1,80) = 0.011, *p* = 0.917
		Sniffing (s)		**F(7,80) = 4.410, *p* < 0.001**	F(1,80) = 0.166, *p* = 0.685	F(1,80) = 0.043, *p* = 0.835	**F(1,80) = 19.626, *p* < 0.001**	F(1,80) = 0.195, *p* = 0.660	F(1,80) = 4.988, *p* = **0.028**	**F(1,80) = 5.299, *p* = 0.024**	F(1,80) = 1.127, *p* = 0.292
	10W	Duration (s)	Three-way ANOVA	**F(11,102)=21.618, *p* < 0.001**	F(1.102) = 0.000, *p* = 1.000	F(1.102) = 0.000, *p* =1.000	**F(2.102) = 101.6, *p* < 0.001**	F(1.102) = 0.000, *p* = 1.000	**F(2.102) = 5.904, *p* = 0.004**	**F(2.102) = 9.791, *p* < 0.001**	F(2.102) = 0.532, *p* = 0.589
		No. of entries		F(7,68) = 0.609, *p* = 0.746	F(1,68) = 1.049, *p* = 0.309	F(1,68) = 1.152, *p* = 0.287	F(1,68) = 0.370, *p* = 0.545	F(1,68) = 0.039, *p* = 0.844	F(1,68) = 0.130, *p* = 0.719	F(1,68) = 0.064, *p* = 0.801	F(1,68) = 1.058, *p* = 0.307
		Sniffing (s)		**F(7,68) = 9.675, *p* < 0.001**	**F(1,68) = 4.128, *p* = 0.046**	F(1,68) = 3.453, *p* = 0.067	**F(1,68) = 26.493, *p* < 0.001**	F(1,68) = 0.130, *p* = 0.720	**F(1,68) = 13.125, *p* = 0.001**	**F(1,68) = 14.596, *p* < 0.001**	**F(1,68) = 4.869, *p* = 0.031**
Social interaction			Two-way ANOVA	F(3,57) = 6.207, *p* = 0.01	Genotype			Sex			Genotype × Sex
					**F(1,57) = 14.966, *p* < 0.001**			F(1,57) = 1.120, *p* = 0.294			F(1,57) = 1.891, *p* = 0.174
Tube test			Binomial test		Male WT *vs*. KO						Female WT *vs*. KO
					p = 1.000 (Win, WT : KO = 6 : 6)						*p* = 0.815 (Win, WT : KO = 8 : 10)
Food exploring					Genotype			Sex			Genotype × Sex
	7W		Two-way ANOVA	F(3,54) = 1.785, *p* = 0.161	F(1,54) = 1.874, *p* = 0.177			F(1,54) = 3.416, *p* = 0.070			F(1,54) = 0.207, *p* = 0.551
	11W			F(3,47) = 0.961, *p* = 0.419	F(1,47) = 0.154, *p* = 0.697			F(1,47) = 0.505, *p* = 0.481			F(1,47) = 2.493, *p* = 0.121
Hot plate			Two-way ANOVA	F(3,83) = 0.869, *p* = 0.461	F(1,83) = 2.119, *p* = 0.149			F(1,83) = 0.240, *p* = 0.625			F(1,83) = 0.186, *p* = 0.668
UV experiment	Bedding		Three-way ANOVA	**F(7,98) = 3.336, *p* = 0.003**	Genotype	Sex	Bedding	Genotype × Sex	Genotype × Bedding	Sex × Bedding	Genotype × Sex × Bedding
					**F(1,98) = 5.357, *p* = 0.023**	F(1,98) = 0.001, *p* = 0.976	**F(1,98) = 10.577, *p* = 0.002**	**F(1,98) = 4.129, *p* = 0.045**	**F(1,98) = 5.313, *p* = 0.023**	F(1,98) = 0.000, *p* = 0.991	F(1,98) = 0.037, *p* = 0.848
	Temperature		Three-way ANOVA	**F(11,128) = 3.931, *p* < 0.001**	Genotype	Sex	Temperature	Genotype × Sex	Genotype × Temperature	Sex × Temperature	Genotype × Sex × Temperature
					F(1,128) = 0.279, *p* = 0.599	F(1,128) = 0.367, *p* = 0.546	**F(2,128) = 8.288, *p* < 0.001**	F(1,128) = 0.426, *p* = 0.515	**F(2,128) = 7.243, *p* = 0.001**	F(2,128) = 1.689, *p* = 0.189	**F(2,128) = 4.827, *p* = 0.010**

Supplementary Table S2.
Statistical *p*-values from multiple comparisons (PLSD, Bonferroni, Sidak, and Tukey HSD) for behavioural studies. Significant results are indicated in bold.

Test		Subject	Comparison	*p*-values from multiple comparison			
				PLSD	Bonferroni	Sidak	Tukey HSD
Three chamber test (6W)	Duration	Male KO	Stranger *vs*. Object	**0.001**	**0.034**	**0.033**	**0.025**
	Sniffing	Male WT	Stranger *vs*. Object	**0.008**	0.226	0.203	0.133
		Male KO	Stranger *vs*. Object	0.560	1.000	1.000	0.999
		Female WT	Stranger *vs*. Object	**<0.001**	**0.009**	**0.009**	**0.007**
		Female KO	Stranger *vs*. Object	**0.005**	0.131	0.123	0.084
Three chamber test (10W)	Duration	Male KO	Stranger *vs*. Object	**0.009**	0.577	0.440	0.255
		Female WT	Stranger *vs*. Object	**<0.001**	**0.009**	**0.009**	**0.007**
	Sniffing	Male WT	Stranger *vs*. Object	**<0.001**	**0.012**	**0.012**	**0.010**
		Male KO	Stranger *vs*. Object	**0.041**	1.000	0.692	0.438
		Female WT	Stranger *vs*. Object	**<0.001**	**<0.001**	**<0.001**	**<0.001**
		Female KO	Stranger *vs*. Object	**<0.001**	**0.003**	**0.003**	**0.003**
Social interaction test		Male	WT *vs*. KO	**<0.001**	**0.002**	**0.002**	**0.002**
		Female	WT *vs*. KO	0.093	0.556	0.442	0.328
UV experiment	Bedding	Male WT	Familiar *vs*. Unfamiliar	**0.015**	0.433	0.354	0.223
		Male KO	Familiar *vs*. Unfamiliar	0,560	1.000	1.000	0.999
		Female WT	Familiar *vs*. Unfamiliar	**0.007**	0.188	0.172	0.115
		Female KO	Familiar *vs*. Unfamiliar	0.671	1.000	1.000	1.000
		Male Unfamiliar	WT *vs*. KO	**0.002**	0.061	0.059	**0.044**
	Temperature	Male WT	19℃ *vs*. 9℃	**<0.001**	**<0.001**	**<0.001**	**<0.001**
		Male KO	19℃ *vs*. 9℃	**0.037**	1.000	0.916	0.617
		Female WT	19℃ *vs*. 9℃	**0.004**	0.240	0.213	0.132
		Female KO	19℃ *vs*. 9℃	**0.026**	1.000	0.822	0.513
		Male 9℃	WT *vs*. KO	**0.001**	0.078	0.075	0.051
		KO 9℃	Male *vs*. Female	**0.011**	0.745	0.527	0.308

Supplementary Table S3.
Statistical p-values from multiple comparison (PLSD, Bonferroni, Sidak, and Tukey HSD) for gene expression analysis. Significant results are indicated in bold.

Brain region	Gene	Comparison	*p*-values from multiple comparison			
			PSLD	Bonferroni	Sidak	Tukey HSD
OB	*GluR2*	Female WT *vs*. KO	**0.019**	0.115	0.110	0.079
	VGluT1	Male WT *vs*. KO	**0.010**	0.057	0.056	**0.041**
		Female WT *vs*. KO	**<0.001**	**0.001**	**0.001**	**0.001**
		WT male *vs*. female	**0.003**	**0.021**	**0.020**	**0.016**
	VGluT2	KO male *vs*. female	**0.014**	0.087	0.083	0.061
	GABAAα1	Female WT *vs*. KO	**<0.001**	**<0.001**	**<0.001**	**<0.001**
		KO male *vs*. female	**<0.001**	**<0.001**	**<0.001**	**<0.001**
	GABAAγ2	Female WT *vs*. KO	**<0.001**	**0.001**	**0.001**	**0.001**
		KO male *vs*. female	**0.006**	**0.039**	**0.038**	**0.029**
	GABABR1	Female WT *vs*. KO	**0.002**	**0.012**	**0.012**	**0.009**
		KO male *vs*. female	**0.029**	0.174	0.162	0.114
	VGAT	Female WT *vs*. KO	**<0.001**	**0.003**	**0.003**	**0.002**
		KO male *vs*. female	**0.012**	0.073	0.071	0.052
	Ncam1	Male WT *vs*. KO	**0.009**	0.053	0.051	**0.038**
		Female WT *vs*. KO	**0.007**	**0.039**	**0.039**	**0.029**
Hippocampus	*GluR1*	Male WT *vs*. KO	**0.014**	0.082	0.079	0.057
		KO male *vs*. female	**0.015**	0.088	0.085	0.062
	GluR2	Male WT *vs*. KO	**0.015**	0.092	0.089	0.063
	GABAAα1	KO male *vs*. female	**0.031**	0.186	0.172	0.121
	GABABR1	KO male *vs*. female	**0.034**	0.204	0.187	0.132
Cortex	*GABAAγ2*	Feale WT *vs*. KO	**0.003**	**0.016**	**0.016**	**0.012**
	GABABR1	WT male *vs*. Female	**0.012**	0.072	0.070	0.051
		Male WT *vs*. KO	**0.014**	0.083	0.080	0.058
	Ncam1	Male WT *vs*. KO	**0.003**	**0.020**	**0.019**	**0.015**
		Female WT *vs*. KO	**0.002**	**0.010**	**0.010**	**0.008**
	N-cadherin	Male WT *vs*. KO	**0.002**	**0.012**	**0.012**	**0.009**
		Female WT *vs*. KO	**<0.001**	**<0.001**	**<0.001**	**<0.001**

Supplementary Table S4. Gene expressions without significant differences among groups in adults

Brain region	Genes	*F*-statistics, degree of freedom, and *p*-values for two-way ANOVA						Expression levels			
			For each factor and interaction			Fold differences		Male		Female	
			Genotype	Sex	Genotype × Sex	WT *vs.* *Crmp4*-KO (*Crmp4*-KO/WT)	Male *vs.* Female (Female/Male)	WT	*Crmp4*-KO	WT	*Crmp4*-KO
Olfactory bulb	*Crmp4*	Not performed				Not performed	1.102	2.578 ± 0.145	ND	2.958 ± 0.099	ND
	GluR1	F(3,12) = 0.131, *p* = 0.940	F(1,12) = 0.218, *p* = 0.649	F(1,12) = 0.173, *p* = 0.685	F(1,12) = 0.001, *p* = 0.976	1.102	1.090	0.861 ± 0.255	0.959 ± 0.040	0.949 ± 0.211	1.034 ± 0.205
	D1R	F(3,8) = 1.166, *p* = 0.381	F(1,8) = 3.097, *p* = 0.116	F(1,8) = 0.003, *p* = 0.961	F(1,8) = 0.398, *p* = 0.546	1.895	1.018	0.263 ± 0.118	0.395 ± 0.178	0.195 ± 0.068	0.474 ± 0.064
	D2R	F(3,8) = 0.871, *p* = 0.495	F(1,8) = 0.236, *p* = 0.640	F(1,8) = 2.369, *p* = 0.162	F(1,8) = 0.007, *p* = 0.938	1.144	1.542	0.300 ± 0.084	0.346 ± 0.018	0.466 ± 0.206	0.531 ± 0.049
	$5HT_{1A}$	F(3,12) = 0.625, *p* = 0.612	F(1,12) = 0.847, *p* = 0.375	F(1,12) = 0.684, *p* = 0.424	F(1,12) = 0.344, *p* = 0.569	1.277	0.804	0.550 ± 0.143	0.789 ± 0.236	0.511 ± 0.100	0.564 ± 0.123
	$5HT_{2A}$	F(3,12) = 1.418, *p* = 0.286	F(1,12) = 0.017, *p* = 0.898	F(1,12) = 2.742, *p* = 0.124	F(1,12) = 1.494, *p* = 0.245	0.938	2.374	0.110 ± 0.067	0.295 ± 0.115	0.596 ± 0.302	0.368 ± 0.070
	$5HT_{7}$	F(3,8) = 0.143, *p* = 0.474	F(1,8) = 1.617, *p* = 0.239	F(1,8) = 4.821, *p* = 0.059	F(1,8) = 0.772, *p* = 0.405	1.425	1.869	0.612 ± 0.111	0.716 ± 0.017	0.959 ± 0.102	1.524 ± 0.503
	GABAAβ2	F(3,12) = 1.629, *p* = 0.235	F(1,12) = 2.730, *p* = 0.124	F(1,12) = 0.215, *p* = 0.651	F(1,12) = 1.942, *p* = 0.189	1.216	1.056	2.784 ± 0.302	2.872 ± 0.317	2.464 ± 0.481	3.509 ± 0.218
Hippocampus	*Crmp4*	Not performed				Not performed	0.879	0.700 ± 0.060	ND	0.615 ± 0.042	ND
	VGluT2	F(3,12) = 0.666, *p* = 0.589	F(1,12) = 0.936, *p* = 0.352	F(1,12) = 0.901, *p* = 0.361	F(1,12) = 0.161, *p* = 0.695	0.847	1.175	0.779 ± 0.056	0.700 ± 0.077	0.963 ± 0.196	0.775 ± 0.164
	D1R	F(3,9) = 3.546, *p* = 0.061	F(1,9) = 3.611, *p* = 0.090	F(1,9) = 3.156, *p* = 0.109	F(1,9) =4.288, *p* = 0.068	1.290	0.789	0.674 ± 0.118	0.660 ± 0.044	0.368 ± 0.016	0.683 ± 0.014
	D2R	F(3,8) = 0.215, *p* = 0.883	F(1,8) = 0.216, *p* = 0.655	F(1,8) = 0.176, *p* = 0.686	F(1,8) = 0.253, *p* = 0.629	1.169	1.153	0.132 ± 0.053	0.130 ± 0.009	0.128 ± 0.016	0.174 ± 0.079
	$5HT_{1A}$	F(3,12) = 1.365, *p* = 0.301	F(1,12) = 2.665, *p* = 0.129	F(1,12) = 1.424, *p* = 0.256	F(1,12) = 0.005, *p* = 0.943	1.303	1.213	0.836 ± 0.303	1.326 ± 0.228	1.241 ± 0.170	1.602 ± 0.170
	$5HT_{2A}$	F(3,11) = 1.657, *p* = 0.233	F(1,11) = 0.059, *p* = 0.813	F(1,11) = 4.094, *p* = 0.068	F(1,11) = 0.512, *p* = 0.489	1.117	0.377	0.136 ± 0.062	0.183 ± 0.067	0.071 ± 0.038	0.048 ± 0.019
	$5HT_{7}$	F(3,8) = 1.211, *p* = 0.366	F(1,8) = 0.781, *p* = 0.403	F(1,8) = 1.004, *p* = 0.346	F(1,8) = 1.849, *p* = 0.211	1.373	1.435	0.297 ± 0.057	0.241 ± 0.085	0.255 ± 0.061	0.517 ± 0.200
	GABAAβ2	F(3,12) = 0.416, *p* = 0.745	F(1,12) = 0.216, *p* = 0.650	F(1,12) = 0.992, *p* = 0.339	F(1,12) = 0.040, *p* = 0.845	0.916	1.223	0.281 ± 0.012	0.244 ± 0.044	0.328 ± 0.095	0.314 ± 0.045
	GABAAγ2	F(3,12) = 0.396, *p* = 0.758	F(1,12) = 0.475, *p* = 0.504	F(1,12) = 0.357, *p* = 0.561	F(1,12) = 0.357, *p* = 0.561	1.096	0.917	0.193 ± 0.012	0.229 ± 0.042	0.193 ± 0.026	0.194 ± 0.020
	VGAT	F(3,12) = 0.503, *p* = 0.688	F(1,12) = 0197, *p* = 0.665	F(1,12) = 0.911, *p* = 0.359	F(1,12) = 0.401, *p* = 0.539	1.047	0906	0.302 ± 0.017	0.296 ± 0.053	0.255 ± 0.016	0.287 ± 0.014
	Ncam1	F(3,12) = 1.535, *p* = 0.256	F(1,12) = 2.512, *p* = 0.139	F(1,12) = 2.077, *p* = 0.175	F(1,12) = 0.014, *p* = 0.906	1.084	1.076	0.082 ± 0.041	0.141 ± 0.071	1.035 ± 0.159	1.318 ± 0.223
Cortex	*Crmp4*	Not performed				Not performed	1.182	0.615 ± 0.059	ND	0.727 ± 0.058	ND
	GluR1	F(3,8) = 2.287, *p* = 0.155	F(1,8) = 0.547, *p* = 0.481	F(1,8) = 4.394, *p* = 0.069	F(1,8) = 1.921, *p* = 0.203	0.854	1.574	0.294 ± 0.031	0.162 ± 0.023	0.339 ± 0.056	0.379 ± 0.104
	GluR2	F(3,10) = 1.874, *p* = 0.198	F(1,10) = 0.004, *p* = 0.948	F(1,10) = 1.455, *p* = 0.255	F(1,10) = 3.410, *p* = 0.095	1.006	0.860	0.392 ± 0.059	0.319 ± 0.019	0.267 ± 0.015	0.345 ± 0.035
	VGluT1	F(3,12) = 1.719, *p* = 0.216	F(1,12) = 1.631, *p* = 0.226	F(1,12) = 2.892, *p* = 0.115	F(1,12) = 0.663, *p* = 0.442	1.004	1.011	1.085 ± 0.008	1.084 ± 0.004	1.091 ± 0.009	1.101 ± 0.004
	D1R	F(3,8) = 0.403, *p* = 0.755	F(1,8) = 0.000, *p* = 0.985	F(1,8) = 0.048, *p* = 0.833	F(1,8) = 1.163, *p* = 0.312	1.005	0.943	1.932 ± 0.451	1.467 ± 0.476	1.362 ± 0.151	1.845 ± 0.567
	D2R	F(3,8) = 1.369, *p* = 0.320	F(1,8) = 0.396, *p* = 0.547	F(1,8) = 1.701, *p* = 0.228	F(1,8) = 2.009, *p* = 0.194	1.288	1.709	0.746 ± 0.034	0.483 ± 0.207	0.709 ± 0.242	1.393 ± 0.587
	$5HT_{1A}$	F(3,8) = 1.789, *p* = 0.227	F(1,8) = 0.143, *p* = 0.715	F(1,8) = 1.520, *p* = 0.253	F(1,8) = 3.703, *p* = 0.091	0.936	1.241	1.010 ± 0.071	1.369 ± 0.158	1.742 ± 0.407	1.208 ±0.138
	$5HT_{2A}$	F(3,8) = 0.815, *p* = 0.521	F(1,8) = 1.502, *p* = 0.255	F(1,8) = 0.944, *p* = 0.360	F(1,8) = 0.000, *p* = 0.984	1.367	0.779	0.168 ± 0.029	0.222 ± 0.014	0.126 ± 0.023	0.179 ± 0.079
	$5HT_{7}$	F(3,8) = 0.028, *p* = 0.993	F(1,8) = 0.021, *p* = 0.888	F(1,8) = 0.043, *p* = 0.841	F(1,8) = 0.021, *p* = 0.889	0.977	0.969	0.953 ±0.105	0.953 ± 0.111	0.944 ± 0.231	0.902 ± 0.096
	GABAAα1	F(3,12) = 1.880, *p* = 0.187	F(1,12) = 3.673 *p* = 0.079	F(1,12) = 1.198, *p* = 0.295	F(1,12) = 0.770, *p* = 0.398	0.784	0.874	0.169 ±0.029	0.149 ± 0.016	0.165 ± 0.014	0.113 ± 0.008
	GABAAβ2	F(3,12) = 0.792, *p* = 0.521	F(1,12) = 0.749, *p* = 0.404	F(1,12) =1.489, *p* = 0.246	F(1,12) = 0.138, *p* = 0.717	0.830	1.297	0.077 ± 0.017	0.068 ± 0.013	0.105 ± 0.026	0.083 ± 0.010
	VGAT	F(3,12) = 2.826, *p* = 0.084	F(1,12) = 3.913, *p* = 0.071	F(1,12) = 1.120, *p* = 0.311	F(1,12) =3.445, *p* = 0.088	0.808	1.121	0.597 ± 0.039	0.383 ± 0.026	0.553 ± 0.062	0.546 ± 0.080
Raphe	*SERT*	F(3,10) = 1.175, *p* = 0.911	F(1,10) = 0.437, *p* = 0.523	F(1,10) = 0.062, *p* = 0.808	F(1,10) = 0.063, *p* = 0.807	1.251	0.918	0.438 ± 0.070	0.590 ± 0.109	0.438 ± 0.093	0.506 ± 0.183

Supplementary Table S5. Real-time qRT-PCR primer sequences

Target genes	Primer sequence (5′-3′)		Product size (bp)
	Forward	Reverse	
Crmp4	GGTACAGAGCCTCAGCAAGG	TTATCCCCATTTCCAGCATC	200
VGluT1	TGGGTTTCTGTATCAGCTTTG	TGTGCTGTTGTTGACCATGGACACG	74
VGluT2	CGTGAAGAATGGCAGTATGTCTTC	TGAGGCAAATAGTGCATAAAATATGACT	81
GluR1	CCCTTTACAACGTGGAGGAA	GAACAAGGGCGTCTCTTCTG	152
GluR2	ACGAGTGGCACACTGAGGAA	GCACCCAAGGAAAACCAGAG	103
GAD67	CTCAGGCTGTATGTCAGATGTTC	AAGCGAGTCACAGAGATTGGTC	111
VGAT	CCATTGGCATCATCGTGTT	CCAGTTCATCATGCAGTGGAA	101
GABAAα1	AAGGACCCATGACAGTGCTC	CAGAGTGCCATCCTCTGTGA	149
GABAAβ2	AACGCCTTCCATCATTGTTC	ATCACCACTCCACGACATCA	148
GABAAγ2	AGTGTTTGGATGGCAAGGAC	AGGAGTCCATTTTGGCAATG	111
GABABR1	TCTGGTTGTGCTCTTTGTGC	TCCTCATTGTTGTTGGTGGA	111
DAT	CCAGCAATTCAGTGATGACATC	CAGCATAGCCGCCAGTACAG	69
D1R	CTCCTGATGGAACACCATTG	GCTTAGCCCTCACGTTCTTG	119
D2R	TGCCATTGTTCTTGGTGTG	AGAGGACTGGTGGGATGTTG	98
$5HT_{1A}$	CAAGACGGTCAAGAAGGTGG	CACTACCTGGCTGACCATTC	101
$5HT_{2A}$	TTCGGGCTACAGGATGATTC	TGATGGTTAGGGGGATGAAA	112
$5HT_{7}$	TGCGGTAAGCACACTACAGC	CACAAAGCCTAGACGGGAAG	135
SERT	TGCCTTTTATATCGCCTCCTAC	CAGTTGCCAGTGTTCCAAGA	123
Ncam1	TGTCAAGTGGCAGGAGATGC	GGCGTTGTAGATGGTGAGGGT	138
N-cadherin	AGCGCAGTCTTACCGAAGG	TCGCTGCTTTCATACTGAACTTT	101
β-actin	GCTACAGCTTCACCACCACA	TCTCCAGGGAGGAAGAGGAT	123
